# Combinatorial Strategies With PD-1/PD-L1 Immune Checkpoint Blockade for Breast Cancer Therapy: Mechanisms and Clinical Outcomes

**DOI:** 10.3389/fphar.2022.928369

**Published:** 2022-07-22

**Authors:** Dan Zheng, Xiaolin Hou, Jing Yu, Xiujing He

**Affiliations:** ^1^ Laboratory of Integrative Medicine, Clinical Research Center for Breast, State Key Laboratory of Biotherapy, West China Hospital, Sichuan University and Collaborative Innovation Center, Chengdu, China; ^2^ Department of Neurosurgery, Hospital of Chengdu University of Traditional Chinese Medicine, Chengdu, China

**Keywords:** anti-PD-1/PD-L1, combination therapy, chemotherapy, irradiation, targeted therapy, adverse events, biomarkers

## Abstract

As an emerging antitumor strategy, immune checkpoint therapy is one of the most promising anticancer therapies due to its long response duration. Antibodies against the programmed death-1 (PD-1) and programmed death ligand-1 (PD-L1) axis have been extensively applied to various cancers and have demonstrated unprecedented efficacy. Nevertheless, a poor response to monotherapy with anti-PD-1/PD-L1 has been observed in metastatic breast cancer. Combination therapy with other standard treatments is expected to overcome this limitation of PD-1/PD-L1 blockade in the treatment of breast cancer. In the present review, we first illustrate the biological functions of PD-1/PD-L1 and their role in maintaining immune homeostasis as well as protecting against immune-mediated tissue damage in a variety of microenvironments. Several combination therapy strategies for the combination of PD-1/PD-L1 blockade with standard treatment modalities have been proposed to solve the limitations of anti-PD-1/PD-L1 treatment, including chemotherapy, radiotherapy, targeted therapy, antiangiogenic therapy, and other immunotherapies. The corresponding clinical trials provide valuable estimates of treatment effects. Notably, several combination options significantly improve the response and efficacy of PD-1/PD-L1 blockade. This review provides a PD-1/PD-L1 clinical trial landscape survey in breast cancer to guide the development of more effective and less toxic combination therapies.

## 1 Introduction

Female breast cancer has been ranked the most prevalent diagnosed cancer since 2020, with an estimated 11.7% of new cases among all malignant diseases (Sung et al., 2021). According to the expression status of hormonal receptors (HR) and human epidermal growth factor receptor 2 (HER2), breast cancer is categorized into three major subtypes: HR positive [defined as estrogen receptor (ER)-positive and/or progesterone receptor (PR)-positive]/HER2-negative, HER2-positive (HR positive or negative), and triple-negative breast cancer (TNBC, defined as ER-negative, PR-negative, and HER2-negative) (Waks and Winer, 2019). The HR-positive/HER2-negative subtype accounts for 70% of breast cancer, while the HER2-positive and TNBC subtypes comprise 20%–25% and 15%–20% of breast cancer, respectively (Waks and Winer, 2019).

Generally, the treatment regimen of breast cancer is mainly combining surgery with chemotherapy, and on this basis, the corresponding specific drugs, including endocrine therapy and anti-HER2 treatment, are added according to the expression status of HR and HER2. However, approximately 20%–30% of breast cancers will eventually relapse and need further treatment in the recurrence or metastasis phase (Liang et al., 2020). For HR-positive and HER2-positive local advanced/metastatic breast cancer, cyclin-dependent kinase 4/6 (e.g., palbociclib) inhibitors and anti-HER2 drugs are the backbone of treatment strategies, respectively (Gradishar et al., 2021; Loibl et al., 2021). Although under standard treatments, almost all these patients suffer from disease progression, and no efficacious strategy is available to control it. Moreover, it is difficult to cope with TNBC due to its heavy heterogeneity (Yin et al., 2020).

Immunotherapy, especially immune checkpoint blockade (ICB), has achieved great success in several kinds of solid tumors but a poor response to single drug has been observed in breast cancer (Keenan and Tolaney, 2020; Esteva et al., 2019). To date, a total of 12 kinds of ICB have been approved for cancer immunotherapy, including seven PD-1 inhibitors (nivolumab, pembrolizumab, cemiplimab, camrelizumab, tislelizumab, toripalimab, and sintilimab), three PD-L1 inhibitors (atezolizumab, durvalumab, and avelumab), and two CTLA-4 inhibitors (ipilimumab and tremelimumab) (Kennedy and Salama, 2020). However, only pembrolizumab and atezolizumab have been approved for treating local advanced or metastatic PD-L1-positive TNBC (Kwapisz, 2021).

The traditional concept of TNBC as low-immunogenic breast cancer has changed over the past decade with the development of “omics”, which revealed a high genetic instability in TNBC (Jiang et al., 2019) and made it a potential candidate for ICB therapy. The genomic characteristics of TNBC endow it with a higher propensity to generate neoantigens (Bianchini et al., 2016), thereby inducing a more friendly tumor microenvironment (TME) (Lei et al., 2020), characterized by more tumor-infiltrating T cells and a higher level of PD-L1 expression (Keenan and Tolaney, 2020; Denkert et al., 2018; Loi et al., 2019). Compared to those with wild-type TP53 tumors, patients with TP53-mutated breast tumors demonstrate higher levels of immune infiltration and more active immunity, which further indicates better survival outcomes (Guo et al., 2017; Liu et al., 2019). In addition, another aggressive subtype of breast cancer, HER2-positive breast cancer, has also been reported to have a relatively higher tumor mutation burden and Tumor-infiltrating lymphocytes (TILs) than the luminal subtype (Holgado et al., 2018). However, rare patients with ER-positive breast cancer are likely to benefit from ICBs (Emens, 2018). Therefore, the effects of ICBs have been detected in both TNBC and HER2-positive breast cancers (De Melo Gagliato et al., 2017).

Although excellent tumor control effects have been observed in other solid tumors, the efficacy of anti-PD-1/PD-L1 monotherapy in breast cancer was disappointing in many clinical trials (Emens, 2018). Atezolizumab demonstrated an overall response rate (ORR) of only 10% in metastatic TNBC (mTNBC) patients who were unscreened for PD-L1 expression status (Emens, 2018). Although KEYNOTE-012 reported an objective response rate (ORR) of only 18.5% in both chemotherapeutic pretreated and naïve PD-L1-positive mTNBC patients (Nanda et al., 2016), KEYNOTE-086 demonstrated a poor ORR of 5.3% in PD-L1-unselected mTNBC patients who were pretreated with chemotherapy (Adams et al., 2019). Therefore, considerable effort has been devoted to developing combinatorial regimens to extend the great potential of ICBs to breast cancer.

**FIGURE 1:**
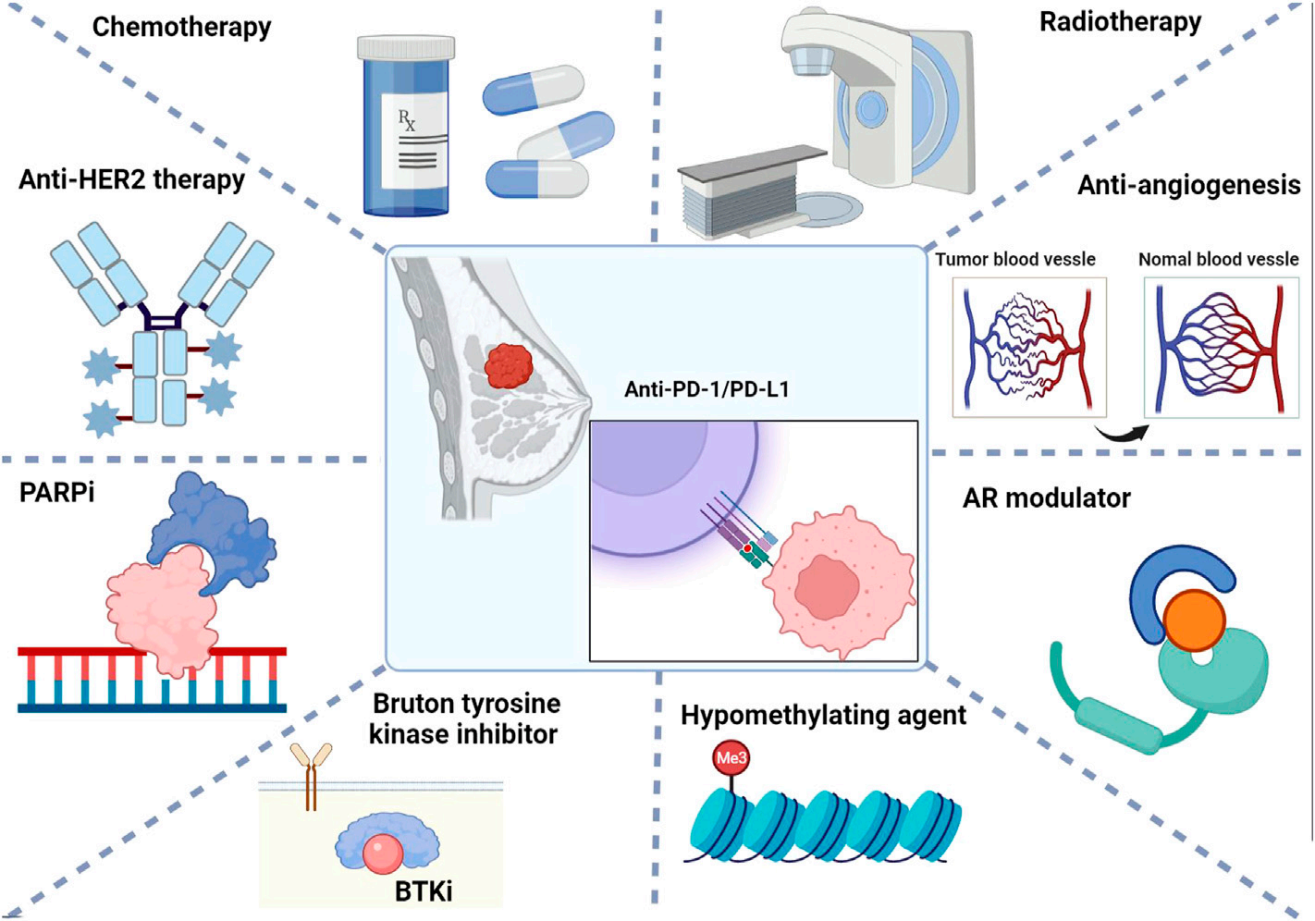
Summary of combinatorial strategies with PD-1/PD-L1 immune checkpoint blockade for breast cancer therapy.

In the present review, we first elaborate on the biological mechanisms underlying ICB to enhance the current understanding of the immune checkpoint molecules PD-1/PD-L1. We then focused on the efficacy, side effects, and molecular biomarkers of the current combinatorial strategies of anti-PD-1/PD-L1 in combination with other local or systematic therapeutic regimens for breast cancer therapy, aiming to expand the ideas for developing more effective and less toxic combinatorial strategies with PD-1/PD-L1 blockade (Figure 1).

## 2 Mechanisms of Anti-PD-1/PD-L1 Therapy

### 2.1 T Cell Activation Procedure

The antitumor immunity effect requires the initiation of adaptive immunity involving T cells. Kevin Lafferty (Lafferty and Prowse, 1984) proposed in his classic dual-signal hypothesis that the activation of T cells requires two steps of antigen recognition and then costimulation (Xu et al., 2016; Sun et al., 2018; Chapman et al., 2020). In this process, the presentation of antigens to effector T cells is steered by antigen-presenting cells (APCs), mainly dendritic cells (DCs). Through T cell receptor (TCR) recognition of the antigen-MHC I complex presented by APCs and costimulatory signals, T cells are activated to proliferate and differentiate into effector T cells and subsequently infiltrate into the TME. Upon activation and infiltration, effector T cells are endowed with a specific weapon to kill tumor cells. Thus, both the disorder of recognition of antigen by the TCR and the attenuation of costimulation signals can impair adaptive immunity.

The engagement of TCR by the antigen-MHC I complex is one of the primary events for T cell activation. First, the antigen is recognized and presented by the MHC-I molecule of APCs to the TCR, whose α- and β-chains are noncovalently associated with the low-molecular-mass transmembrane protein of the CD3 complex (Allison, 1994; Alegre et al., 2001). Once binding occurs, the antigen-MHC complex recruits various signaling molecules, including the src-family kinase Lck and the syk-family kinase ZAP-70, to assist the formation.

### 2.2 Co-Regulatory Signals

Accompanied with the antigen-MHC complex recognition by the TCR, the coexpression of molecules on the surface of tumor cells and T cells generates adjuvant signals (both stimulation and inhibition) to regulate the activation of T cells. Complicated mechanisms are involved in T cell activity regulation, including cell-intrinsic programs, metabolic programs (Maciolek et al., 2014; Yang and Chi, 2012), regulatory T cells (Sakaguchi et al., 2020), and coexpression receptors, which are of critical importance in T cell exhaustion and consequently hinder the adaptive immune cell response. Fortunately, T cell exhaustion has been proven to be reversible (Barber et al., 2006), providing an opportunity to recover the antitumor immune response (Topalian et al., 2012; Wei et al., 2017; Liu and Zheng, 2018).

CD28 is the most effective and best characterized costimulatory molecule that is expressed on the T cell surface and drives critical intracellular biochemical events such as phosphorylative and transcriptional signaling, metabolism, and the production of some cytokines and chemokines to sustain the survival and differentiation of T cells. The survival of T cells is also improved by CD28 signaling through upregulating Bcl-X1, which belongs to the antiapoptotic Bcl-2 family. CD28 transduces signals partially independent of the TCR, while the binding of TCR and antigen is likely to induce apoptosis or anergy of the T cells in the absence of CD28 (Alegre et al., 2001). CD28 generates signals to initiate the activation of T cells and construct tolerance after being triggered by the engagement of its ligands CD80 (B7-1) and CD86 (B7-2) of the B7 protein superfamily, which are expressed on the surface of APCs and T cells.

In the development of malignant diseases, tumor cells impede T cell activation and escape from the elimination of the immune response by expressing the corresponding ligands on their surface to combine with those coinhibitory receptors on the T cells. In addition to CD28, CD80, and CD86 act as ligands for the inhibitory receptor CTLA-4. Upon binding, B7:CD28 and B7:CTLA-4 provide costimulatory and coinhibitory signals, respectively, to T cells, thereby maintaining immune homeostasis or mediating immune disorder (Lindstein et al., 1989; Taube et al., 2012; Esensten et al., 2016; Xu et al., 2016). Similar to CTLA-4, another coreceptor of the CD28 superfamily, PD-1, which is expressed on the surface of T cells, and its ligands PD-L1/PD-L2, which are expressed on the surface of tumor cells, together constitute another critical coinhibitory signaling pathway participating in regulating the adaptive immune response and are completely independent of B7-1: CTLA-4 (Fife and Bluestone, 2008; Gardner et al., 2014). The ligands PD-L1 (B7-H1) and PD-L2 (B7-DC) of PD-1 also belong to the B7 protein superfamily and are commonly expressed on the surface of macrophages and DCs (Latchman et al., 2001), differing in their expression patterns.

PD-1 is a transmembrane protein, the intracellular part of which is composed of the immunoreceptor tyrosine-based inhibitory motif (ITIM) and the immunoreceptor tyrosine-based switch motif (ITSM). SHP-1 and SHP-2 are two phosphatases that can bind to the ITIM and ITSM motifs of PD-1 to downregulate the antigen receptor signal. Upon binding with the antigen-MHC complex, the two intracellular tyrosine kinases of PD-1 are phosphorylated and subsequently bind with the phosphatases SHP-2 or SHP-1. Then, the intermediate signal is dephosphorylated to downregulate the antigen receptor signal (Keir et al., 2008). PD-1 inhibits the antigen receptor only in *cis*, therefore, the special location close to each other of PD-1 and the antigen receptor is critical to the inhibitory function of PD-1 (Bennett et al., 2003). In addition, the PD-1:PD-L1 signal challenges the effect of CD28:B7 on T cells by inhibiting the cell survival factor Bcl-xL and transcription factors, including GATA-3, Tbet, and Eomes, which are associated with effector cell function (Keir et al., 2008).

### 2.3 Regulation of PD-1/PD-L1 Expression

Although PD-1 and CTLA-4 are both inhibitory receptors, they differ in function and expression. Except for T cells, NK cells, and B cells, PD-1 is expressed on the surface of Tregs, NKT cells, activated monocytes, and myeloid DCs, while it is not expressed on the surface of naïve T cells (Keir et al., 2008; Rotte, 2019). Both PD-L1 and PD-L2 are expressed on T and B cells, DCs, and other bone marrow-derived cells, while PD-L1 is additionally expressed on nonhematopoietic cells, and PD-L2 is expressed at a much higher level than PD-L1 on hematopoietic cells. High PD-L1 has been detected in tumor cells, including breast cancer, renal cell carcinoma, colorectal cancer, non-small cell lung cancer, etc., (Thompson et al., 2004) and is associated with a worse prognosis than low PD-L1 expression tumors (Ohaegbulam et al., 2015). Moreover, several other signals, including MER/ERK, MyD88 or TRAF6-mediated signals, PI3K/AKT, and eIF4F, regulate PD-L1 expression levels in a STAT-dependent manner (Liu et al., 2007; Zhang et al., 2017; Cerezo et al., 2018).

Type I/II interferon (IFN) act as the mainly regulation signaling pathway of PD-L1 expression. The expression of PD-L1 is increased when cells are exposed to interferon-γ (IFN-γ), during which the IFN signal promotes the nuclear translocation of phosphorylated STAT dimers by the JAK/STAT pathway. Subsequently, IFN regulatory factor 1 (IRF1) expression is increased, which in turn upregulates the expression level of PD-L1 (Vranic et al., 2021).

Due to the reversibility of T cell exhaustion and functional inhibition, as well as the clear function of blocking the coreceptors expressed on the cell surface in the recovery of T cell activity, a variety of immunotherapy drugs targeting coreceptors have been developed. As among all kinds of immunotherapies, targeting inhibitory immune checkpoint receptors, especially PD-1, PD-L1, and CTLA-4, is the kind of ICB that most clearly defines regulatory mechanisms and achieves the most success in clinical (Zou et al., 2016). In the past few years, ICB has demonstrated remarkable antitumor effects in several solid tumors, especially in tumors with positive PD-L1 expression, inspiring the passion to develop ICB treatment strategies for breast cancer (Hu et al., 2017; Emens, 2018; Zhu et al., 2021b).

## 3 Current View and Dilemma of Immunotherapy in Breast Cancer

Although TNBC and HER2-positive breast cancer are moderately immunogenetic, their response to ICBs is poor partly due to the low positive expression rate of PD-L1 in breast cancer. Besides, the low immunogenicity of tumors, hypoxic TME, and several other reasons have been reported to correlate with nonresponse to ICB (Topalian et al., 2016). PD-L1 is expressed in only approximately 20% of TNBC patients (Mittendorf et al., 2014) and 18% of HER2-positive breast cancer patients (Hou et al., 2018), and approximately 50%–80% of patients with tumors show a limited response to ICB monotherapy, with no clinical benefit (Fukumura et al., 2018). Attempts to improve the effect of ICBs in breast cancer have been made over the past few years, involving identifying the potential beneficiary and reforming therapy strategies.

Previous clinical studies reported the clinical outcomes of metastatic breast cancer patients under treatment with a single ICB drug. The phase I trial of PCD4989g (NCT01375842) reported a median progression-free survival (mPFS) of 1.4 months and a median overall survival (mOS) of 8.9 months (Emens et al., 2019), which was similar to the outcomes reported by trial KEYNOTE-012 (NCT02447003) (Nanda et al., 2016). In addition, pembrolizumab monotherapy did not show better efficacy than single-agent chemotherapy in the clinical trial of KEYNOTE-119 (Winer et al., 2021).

Therefore, the focus has shifted from monotherapy of ICB to combination with other therapeutics, including chemotherapy and irradiation, to promote the release of tumor-associated antigens (TAAs), improve immunogenicity, and combine with antiangiogenic drugs to attenuate the hypoxic TME (Zhao et al., 2019; Yi et al., 2022).

**FIGURE 2:**
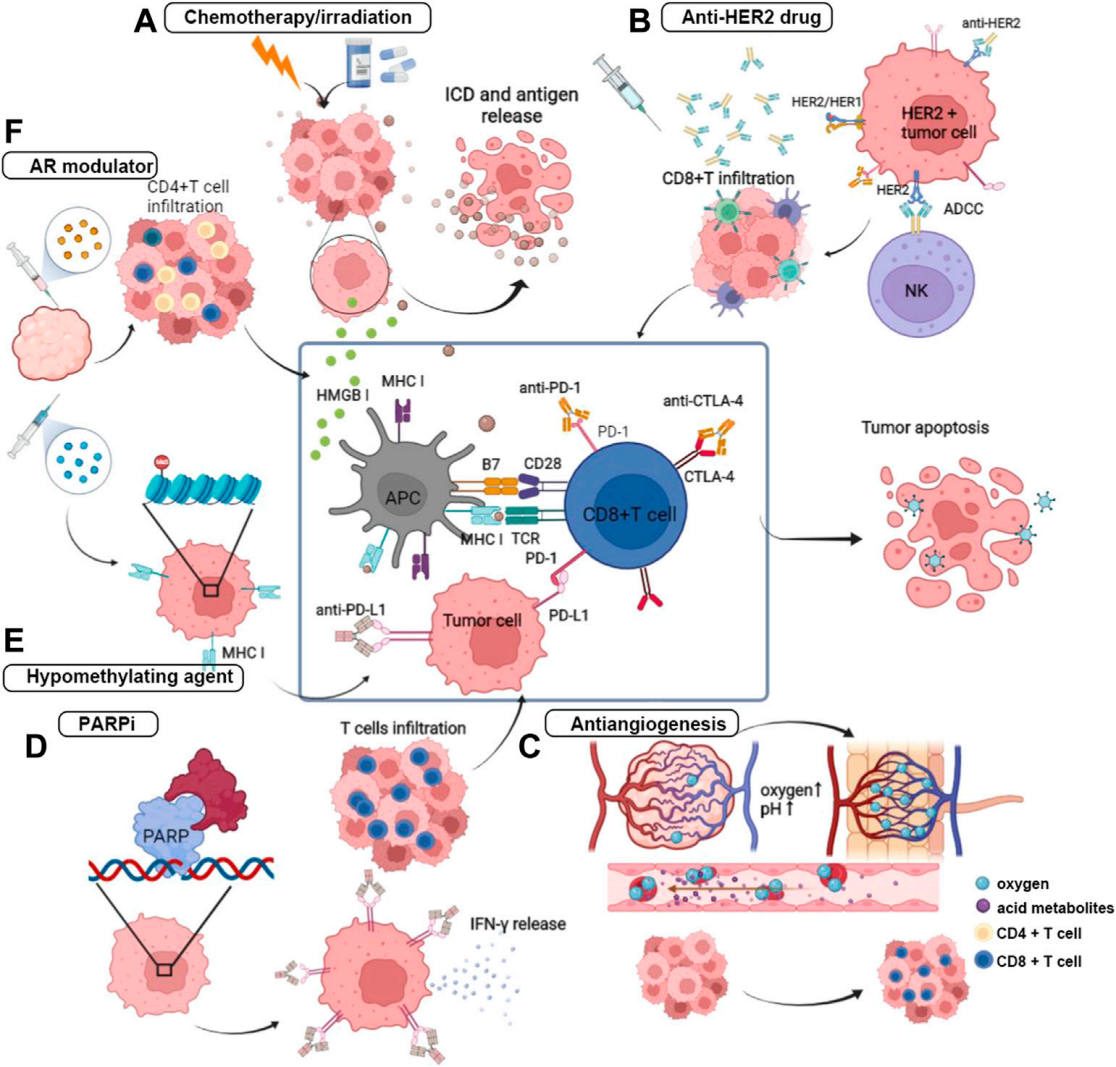
Anti-tumor mechanisms of PD-1/PD-L1 blockade combining with other treatment regimens. **(A)** The primary mechanism of chemotherapy and irradiation in combination with ICB is to induced ICD, and thereby to promote the release of tumor antigen. Under the promotion of ICD, HMGB I translocated to improve antigen presentation and the following process of T cell activation. **(B)** The Fc structure of the monoclonal antibody of HER2 mediates the ADCC by binding with Fc receptor on the NK cells and promote the CD8-positive T cells infiltration. **(C)** Anti-angiogenic therapy acts on reshaping the disorder blood vessel and attenuating the hypoxic and acid TME. **(D)** PARPi inhibits the DNA damage repair by impeding the binding of PAPR on the broken DNA, and then induces the activation of IFN pathway and effector cells infiltration. **(E)** Hypomethylating agent prompts antigen presentation by upregulating the expression of MHC class I. **(F)** Androgen receptor antagonist inhibits the IL-10 release and T_reg_ infiltrartion, and attracts effector T cells further.

Beyond the role of directly killing cancer cells, chemotherapy positively regulates the immune system by modifying the TME, which is greatly conducive to the anticancer effect of immunotherapy. Given the property of chemotherapy in coordinating the cancer immune response, strategies of PD-1/PD-L1 blockade in combination with chemotherapeutic drugs have been designed in clinical trials and has achieved remarkable clinical outcomes. The most common pathway by which chemotherapy improves the response of tumor cells to ICB is to induce immunogenic cell death (ICD) and subsequently promote the release of TAAs (Kono et al., 2013; Inoue and Tani, 2014). Chemotherapeutic drugs, including alkylating agents and anti-tubulin agents, have been proven efficient in arousing ICD (Wu and Waxman, 2018). During this process, the ER chaperone protein calreticulin carries an “eat-me” signal and translocates to the cell surface, which facilitates the engulfment of DCs and tumor antigen uptake. Upon stimulation of ICD, high mobility group box 1 (HMGB1) protein is exported from the nucleus, mounted on the cellular membrane, and then released to interact with toll-like receptor 4 (TLR4) of DCs to promote the antigen presentation process and CD8+ T cell activation (Apetoh et al., 2007; Zitvogel et al., 2008). Furthermore, chemotherapy improves the immunogenicity of tumor cells by upregulating MHC-I and tumor-specific antigens on the cell surface and activating NK cells by inducing the expression of their stimulatory ligands (Wu and Waxman, 2018) (Figure 2).

There is also a high interest in synergizing irradiation with immunotherapy, as substantial evidence has shown that ionizing radiation improves by promoting tumor antigen release and presentation (Golden and Apetoh, 2015; Zhu et al., 2021a). In addition, damage-associated molecular patterns (DAMPs) derived from ICD and cytokines can participate in immune cell recruitment and promote the function of DCs (Yi et al., 2022). Studies have also found irradiation-induced shrinkage of tumors both within local lesions and at distances, which was called the “abscopal effect” (Yi et al., 2022). This phenomenon indicates an immune activation function of irradiation, which provides a basis for combining with ICB (Rodriguez-Ruiz et al., 2018) (Figure 2).

HER2-positive breast cancer is intrinsically invasive, with HER2 overexpressed on the cell surface. HER2 belongs to the epidermal growth factor receptor family and can be activated through self-dimerization or with other HER2 family receptors due to a lack of ligands. After the first anti-HER2 humanized IgG1 monoclonal antibody, trastuzumab being approved, the prognosis of this disease was significantly improved because the blockade of HER2 receptor dimerization inhibited the activation of this signaling pathway (Baselga et al., 2012). Trastuzumab can block HER2 signaling and activate the antitumor immune response depending on the activation of antibody-binding Fc receptors (FcRs) to destroy tumor cells (Gennari et al., 2004; Spector and Blackwell, 2009; Stagg et al., 2011). Research revealed that anti-HER2 monoclonal antibody (mAb) are unable to kill tumor cells when not bound to FcR, and patients treated with trastuzumab showed an increase in FcR^+^ cell infiltration (Arnould et al., 2006; Varchetta et al., 2007). In addition, the tumor clearance effect of anti-HER2 mAb has also been reported to depend on adaptive immunity (Park et al., 2010). Anti-HER2 monoclonal antibody (mAb) has been proven to trigger MyD88-dependent signaling and induce CD8+ T cells to produce INF-γ (Stagg et al., 2011). Therefore, it is reasonable to combine immunotherapy and anti-HER2 drugs to achieve a synergistic function (Figure 2). Currently, two categories of anti-HER2 drugs have been approved in breast cancer including monoclonal antibody, which is represented by trastuzumab and pertuzumab, and antibody-drug conjugates (ADC), which is represented by T-DM1 (ado-trastuzumab emtansine).

Currently, the application of PD-1/PD-L1 blockades combined with novel targeted regimens in the treatment of breast cancer is in its infancy, and the specific mechanisms underlying synergistic or antagonistic effects need further investigation. The morphologically and functionally abnormal tumor vessels establish a hostile TME characterized by local hypoxia, low pH, and elevated interstitial fluid pressure. Anti-angiogenic drugs can remodel the TME by promoting blood vessel normalization, improving T cell infiltration and DC maturation, and simultaneously alleviating the activities of immunosuppressive cells such as myeloid-derived suppressor cells (MDSCs) and Tregs (Ribatti et al., 2019). Considering the ability of antiangiogenic drugs in TME reprogramming, the antitumor effects of PD-1/PD-L1 blockades can be enhanced by combining with antiangiogenic agents (Fukumura et al., 2018). Thus far, several clinical trials have investigated PD-1/PD-L1 blockade combined with antiangiogenic drugs for breast cancer treatment (Figure 2).

Bruton’s tyrosine kinase (BTK) belongs to the Tec kinase family and participates in B cell receptor (BCR) signal transduction (Pan et al., 2007). The inhibitor of Bruton’s tyrosine kinase has been confirmed to have an antitumor effect in combination with anti-PD-L1, which has been approved in B cell malignant disease treatment. Moreover, DNA hypomethylating agents (HMAs) have been proven to increase the expression of HLA I and cancer testis antigens on the tumor cell surface, and thereby prompt tumor recognition by upregulating a series of immunomodulatory pathway-related genes (Li et al., 2014). Moreover, HMAs also function to upregulate the expression of PD-1 on T cells *via* hypomethylation of the PD-1 promoter, promoting CD4+ T cell/CD8+ T cell activation, immune infiltration, and cytolytic function (Daver et al., 2018; Gonda et al., 2020; Loo Yau et al., 2021). Thus, these properties provide the basis for combining HMAs with ICBs. Androgen deprivation therapy has been observed an immunomodulatory effect in solid tumors (Drake et al., 2005; Gamat and Mcneel, 2017). Androgen was reported to be related to the productivity of antigen in different gender mice immunized with polyvinylpyrrolidone. Testosterone can be converted to several kinds of sex hormones that have immunomodulatory effects. Testosterone or androgen dihydrotestosterone (DHT) is able to improve the release of IL-10 by CD4+ T cells and suppress the immune response (Liva and Voskuhl, 2001). In addition, preclinical evidence showed that the absolute level of peripheral T cells increased under castration treatment in mice. Testosterone was also reported to increase the number of CD4+CD25+Foxp3+ Tregs. Given the theoretical basis, androgen receptor (AR) modulators have been used in combination with immunotherapy.

The BRCA1/2 gene participates in DNA damage repair. Germline BRCA1/2 mutation has been found to be associated with deficient DNA double-strand break (DSB) damage repair capacity, which may subsequently induces genomic instability, causes high histological grade TNBC, and younger age at first diagnosis (Copson et al., 2018). Based on this characteristic of tumor cells that carry the BRCA mutation, poly (ADP-ribose) polymerase inhibitor (PARPi) impedes the recognition of PARP protein to bind with the single-strand break (SSB) of DNA and further hinders the repair mechanism of SSB, which finally induces synthetic lethality (Robson et al., 2017; Slade, 2020). Moreover, evidence has supported that anti-PD-1/PD-L1 is less efficacious in a noninflamed TME due to less lymphocyte infiltration and low PD-L1 expression (Zhao et al., 2019). DNA damage may lead to the activation of interferon genes (STING) simulator and NF-κB signals, which in turn initiate inflammation and immune cell infiltration (Green et al., 2017; Stewart et al., 2018). In addition, BRCA mutation was reported to be correlated with the upregulation of PD-L1 (Gottlieb et al., 2017). On the other hand, PARPi function in activating IFN and recruiting effector T cells (Wang et al., 2019; Wu et al., 2021). Therefore, it is reasonable to combine ICB and PARPi in tumors that carry DNA damage repair defects (Figure 2).

## 4 Clinical Application of PD-1/PD-L1 Blockade-Based Combination Treatment for Breast Cancer

Despite great progress in anti-PD-1/PD-L1 immunotherapy, its application to the treatment of breast cancer, especially TNBC, is a huge challenge due to the limited response rate and rapid emergence of resistance and/or serious adverse events (SAEs). Strikingly, PD-1/PD-L1 blockade combined with other treatment regimens produces a satisfactory outcome by enhancing antitumor activity, overcoming drug resistance, and attenuating adverse reactions (Minn and Wherry, 2016). Numerous clinical trials have been initiated to assess the efficacy and safety of PD-1/PD-L1 blockades in combination with other treatment regimens.

**FIGURE 3:**
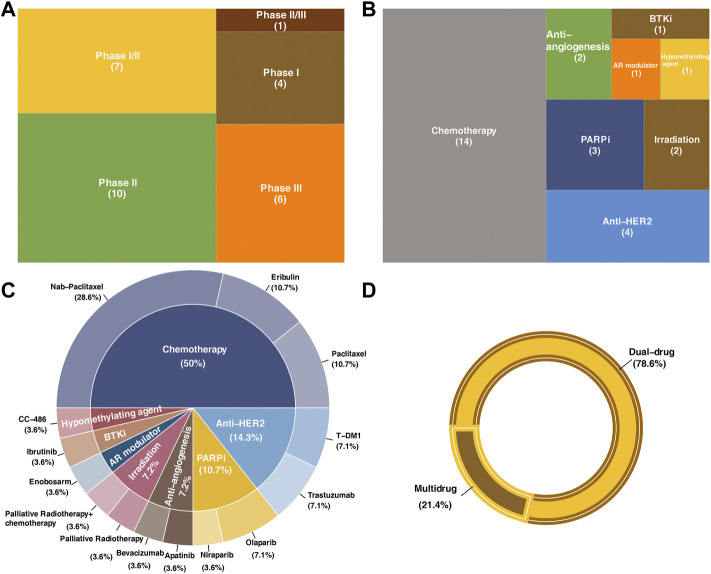
Statistics of clinical trials included in the present review. **(A)** Distribution of the research phase of clinical trials in this review. **(B)** Therapeutic strategies in combination with immune check point blockades. **(C)** Drugs used in each clinical trial and the correspond proportion. **(D)** Proportions of dual-drug strategies and multidrug strategies.

Clinical trials with results retrieved from PubMed, Medline and Clinicaltrials.gov were included and reviewed. In summary, there are a total of 28 trials involving dual-drug therapy, which contain nine chemotherapy combining regimens, two irradiation combining regimens, and four anti-HER2 combining regimens. In addition, clinical trials involving seven other targeted therapy combining regimens, including two antiangiogenic agents, two PARP inhibitors, one hypomethylating agent, one BTK inhibitor, and one AR modulator, were also included. There were six multi-drug therapies, including five chemotherapy combining regimens and one combined with PARPi combining regimen (Table 1). Among all these clinical trials, 78.6% were dual-drug regimens, while the remaining were multidrug regimens that adopted over two kinds of treatments to combine with ICBs (Figure 3).

**TABLE 1:** Clinical trials evaluating PD-1/PD-L1 inhibitors in combination with other therapy strategies in breast cancer.

Combination strategy	NCT	Phase	ICB	Regimen	Combination therapy	ITT
Dual-drug	NCT02628132	I/II	Durvalumab 750 mg, d1, 15	Paclitaxel 80 mg/m2, d1, 8, 15	Chemotherapy	21 Ghebeh et al. (2021)
Dual-drug	NCT03805399	Ib/II	SH1210	Nab-paclitaxel	Chemotherapy	19 Jiang et al. (2021b)
Dual-drug	NCT02513472	Ib/II	Pembrolizumab 200 mg d1, q3w	Eribulin 1.4 mg/m2, d1, 8, q3w	Chemotherapy	167 Tolaney et al. (2021)
Dual-drug	NCT03051659	II	Pembrolizumab 200 mg d1, q3w	Eribulin 1.4 mg/m2, d1, 8, q3w	Chemotherapy	90 Tolaney et al. (2020)
Dual-drug	NCT03222856	II	Pembrolizumab 200 mg d1, q3w	Eribulin 1.23 mg/m2, d1, 8, q3w	Chemotherapy	44 Perez-Garcia et al. (2021)
Dual-drug	NCT02425891	III	Atezolizumab 840 mg d1, 15	Nab-paclitaxel 100 mg/m2, d1, 8, 15	Chemotherapy	902 Schmid et al. (2018)
Dual-drug	NCT03197935	III	Atezolizumab 840 mg d1, 15	Nab-paclitaxel 125 mg/m2, q1w	Chemotherapy	333 Mittendorf et al. (2020)
Dual-drug	NCT03125902	III	Atezolizumab 840 mg d1, 15	Paclitaxel 90 mg/m2, d1, 8, 15	Chemotherapy	651 (Miles et al., 2021)
Dual-drug	NCT01633970	Ib	Atezolizumab 800 mg d1, 15	Nab-paclitaxel 125 mg/m2, d1, 8, 15	Chemotherapy	33 Adams et al. (2019)
Dual-drug	NCT03366844	II	Pembrolizumab 200 mg, d2-7	Palliative radiotherapy 4 Gy × 5	Irradiation	8 Barroso-Sousa et al. (2020b)
Dual-drug	NCT02499367	II	Nivolumab q2w	1)Irradiation 8 Gy × 3, 2)cyclophosphamide 50 mg, qd, 3)cisplatin 40 mg/m2, 4)doxorubcin 15 mg/m2	Irradiation, Chemotherapy	70 Voorwerk et al. (2019)
Dual-drug	NCT02605915	Ib	Atezolizumab 1200 mg	T-DM1 3.6 mg/kg, trastuzumab (6 mg/kg,8 mg/kg), partuzumab (loading 840 mg, maintenance 420 mg), docetaxel 75 mg/m2	Anti-HER2	73 Hamilton et al. (2021)
Dual-drug	NCT02649686	Ib	Durvalumab 1125 mg, d1	Trastuzumab 8 mg/kg loading, followed 6 mg/kg, q3w	Anti-HER2	15 Chia et al. (2019)
Dual-drug	NCT02129556	Ib/II	Pembrolizumab 2 mg/kg, 10 mg/kg, q3w	Trastuzumab 6 mg/kg	Anti-HER2	6 Ib, 52 II Loi et al. (2019)
Dual-drug	NCT02924883	II	Atezolizumab 1200 mg	T-DM1 3.6 mg/kg	Anti-HER2	202
Dual-drug	NCT02802098	I	Durvalumab 10 mg/kg	Bevacizumab 10 mg/kg, q2w	Anti-angiogenesis	26 Quintela-Fandino et al. (2020)
Dual-drug	NCT03394287	II	Camrelizumab 200 mg q2w	Apatinib 250 mg, continuous: d1-14, intermittent: d1-7	TKI	40 Liu et al. (2020)
Dual-drug	NCT02401048	Ib/II	Durvalumab 10 mg/kg	Ibrutinib 560 mg, daily	TKI	45 Hong et al. (2019)
Dual-drug	NCT02811497	II	Durvalumab 1500 mg, d15	CC-486 300 mg, d1–d14, 100 mg, qd, d1–d21	DNA hypomethylating agent	28 Taylor et al. (2020)
Dual-drug	NCT02971761	II	Pembrolizumab 200 mg d1, q3w	Enobosarm 18 mg, daily	Androgen receptor agonist	18 Yuan et al. (2021)
Dual-drug	NCT02734004	I/II	Durvalumab 1500 mg, q4w	Olaparib 300 mg, twice daily	PARPi	30 Domchek et al. (2020)
Dual-drug	NCT02657889	II	Pembrolizumab 200 mg, d1, q3w	Niraparib 200 mg, twice daily	PARPi	55 Vinayak et al. (2019)
Multi-drug	NCT02489448	I/II	Durvalumab 3 mg/kg, 10 mg/kg	Nab-paclitaxel 100 mg/m2-doxorubcin 60 mg/m2 + cyclophosphamide 600 mg/m2	Chemotherapy	45 Ahmed et al. (2020)
Multi-drug	NCT02622074	Ib	Pembrolizumab 200 mg	1)Nab-paclitaxel − doxorubicin + cyclophosphamide 2)nab-paclitaxel + carboplatin − doxorubicin + cyclophosphamide	Chemotherapy	60 Schmid et al. (2020b)
Multi-drug	NCT02685059	II	Durvalumab window: 750 mg, 1500 mg q4w	Nab-paclitaxel 125 mg/m2, weekly, 12w, followed by epirubicin (90 mg/m2) + cyclophosphamide (600 mg/m2)	Chemotherapy	174 Loibl et al. (2019)
Multi-drug	NCT02819518	III	Pembrolizumab	Nab-paclitaxel 100 mg/m2, d1, 8, 15; Paclitaxel 90 mg/m2, d1, 8, 15; or gemcitabine 1,000 mg/m2 plus carboplatin AUC = 2, d1.8	Chemotherapy	847 Cortes et al. (2020)
Multi-drug	NCT03036488	III	Pembrolizumab 200 mg, q3w	Carboplatin AUC 5 + paclitaxel 80 mg/m2, q3w-doxorubcin 60 mg/m2 + cyclophosphamide 600 mg/m2	Chemotherapy	1,174 Schmid et al. (2020a)
Multi-drug	NCT01042379	II/III	Durvalumab	Plaparib + paclitaxel (80 mg/m2) − doxorubicin (60 mg/m2) + cyclophosphamide (600 mg/m2), trastuzumab 4 mg/kg loading, followed 2 mg/kg (for HER2+)	PARPi + chemotherapy	409 Pusztai et al. (2021)

Abbreviations: ITT, intention to treat population.

These clinical trials were categorized and discussed according to the number of drugs within one combination strategy (dual-drug therapy and multidrug therapy) as well as the kind of regimen that the anti-PD-1/PD-L1 immunotherapy combined. The responses were assessed based on the Response Evaluation Criteria in Solid Tumors (RECIST), version 1.1 in all clinical trials, and the severity of adverse events (AEs) was evaluated according to the Common Terminology Criteria for Adverse Events (CTCAE, the version differed in each trial). Key information of these clinical trials is summarized in Table 1. The effects of all clinical trials included in this review are summarized in Table 2.

**TABLE 2:** Clinical trial results of PD-1/PD-L1 blockade in combination with other cancer treatment regimens.

Research	Subtype	Group	PP(n)	Results
NCT02628132 (Ghebeh et al., 2021)	TNBC	Durvalumab + paclitaxel	21	ORR: 26%
				DCR: 47%
				CR: 5%
				mPFS: 4.0 m
				mOS: 20.7 m
NCT03805399 Jiang et al. (2021b)	TNBC	SH1210 + nab-paclitaxel	19	ORR: 62.5%
NCT02513472 Tolaney et al. (2021)	TNBC	Pembrolizumab + eribulin	167	ORR: 25.8 phase I, 21.8% phase II
NCT03051659 Tolaney et al. (2020)	HR(+) HER2(−)	Pembrolizumab + eribulin	44	mOS: 12.5 m
				mPFS: 4.1 m
				ORR: 34.0%
				PR + SD: 70.0%
				DOR: 1.5 m
	HR(+) HER2(−)	Eribulin	46	mOS: 13.4 m
				mPFS: 4.2 m
				ORR: 27.0%
				PR + SD: 70.0%
				DOR: 2.1 m
NCT03222856 Perez-Garcia et al. (2021)	HR(+) HER2(−)	Pembrolizumab + eribulin	44	mPFS: 6.0 m
				1y-OS: 59.1%
				ORR: 40.9%
				DCR: 25%
NCT02425891 Schmid et al. (2018)	TNBC	Atezolizumab + nab-paclitaxel	451	mOS: 21.3 m
				mPFS: 7.2 m
				1y-PFS: 23.7% (19.6–27.9)
				2y-PFS: 42.1% (34.3–49.9), pCR: 7.1%
				ORR: 56.0%
				DOR: 7.4 m
	TNBC	Placebo + nab-paclitaxel	451	mOS: 17.6 m
				mPFS: 5.5 m
				1y-PFS: 17.7% (14.0–21.4)
				2y-PFS: 39.7% (33.2–46.3), pCR: 1.6%
				ORR: 45.9%
				DOR: 5.6 m
NCT03197935 Mittendorf et al. (2020)	TNBC	Atezolizumab + nab-paclitaxel − doxorubicin + cyclophosphamide	165	pCR: 58.0%
	TNBC	Placebo + chemotherapy	168	pCR: 41.0%
NCT03125902 Miles et al. (2021)	TNBC	Atezolizumab + paclitaxel	431	mOS: 19.2 m
				mPFS: 5.9 m
				1y-OS: 69% (64–73)
				2y-OS: 42% (36–48), pCR: 5.0%
				ORR: 43.0%
				DOR: 7.7 m
	TNBC	Placebo + paclitaxel	220	mOS: 22.8 m
				mPFS: 5.6 m
				1y-OS: 73% (67–79)
				2y-OS: 45% (36–54), pCR: 5.0%
				ORR: 36.0%
				DOR: 5.8 m
NCT01633970 Adams et al. (2019)	TNBC	Atezolizumab + nab-paclitaxel	33	ORR: 39.4%
				CR: 3.0%
				DCR: 51.5% mDOR: 9.1 m
				mPFS: 5.5 m
				mOS: 14.7 m
NCT03366844 Barroso-Sousa et al. (2020b)	HR(+) HER2(−)	Pembrolizumab + irradiation	8	mOS: 2.9 m
				mPFS: 1.4 m
NCT02499367 Voorwerk et al. (2019)	TNBC	Nivolumab	12	ORR: 17%
	TNBC	Nivolumab + irradiation	12	ORR: 8%
	TNBC	Nivolumab + cisplatin	13	ORR: 23%
	TNBC	Nivolumab + doxorubicin	17	ORR: 35%
	TNBC	Nivolumab + cyclophosphamide	12	ORR: 8%
NCT02605915 Hamilton et al. (2021)	HER2(+)	Atezolizumab + trastuzumab + pertuzumab	mBC: 6, eBC: 20	ORR: 14% (mBC), 65% (eBC)
	HER2(+)	Atezolizumab + T-DM1	mBC: 6, eBC: 20	ORR: 35% (mBC), 70% (eBC)
	HER2(+)	Atezolizumab + trastuzumab + pertuzumab + doxcetaxel	mBC: 6	ORR: 100% (mBC)
NCT02649686 Chia et al. (2019)	HER2(+)	Durvalumab + trastuzumab	15	SD: 29%
				DOR: 2.7 m mPFS: 1.35 m
				6-month PFS: 0
				6-month OS: 51.6%
				1-year OS: 17.2%
NCT02129556 Loi et al. (2019)	HER2(+)	Pembrolizumab + trastuzumab	58	PD-L1-positive
				ORR: 15%
				DCR: 11% (24w)
				CR: 4%
NCT02924883 Emens et al. (2020)	HER2(+)	Atezolizumab + T-DM1	133	mPFS: 8.2 m
				pCR: 6.0%
				ORR: 45.0%
				DOR: 7.1 m
	HER2(+)	Placebo + T-DM1	69	mPFS: 6.8 m
				pCR: 7.2%
				ORR: 43.0%
				DOR: 9.9 m
NCT02802098 Quintela-Fandino et al. (2020)	HER2(−)	Durvalumablumab + bevacizumab	25	mOS: 11.0 m
				mPFS: 3.5 m
				DCR: 60% (at 8w), 44% (at 16w)
NCT03394287 Liu et al. (2020)	TNBC	Camrelizumab + apatinib continuous	30	mOS: 8.1 m
				mPFS: 3.7 m
				1y-OS: 42.2% (24.2–59.2)
				ORR: 43.3%
				DCR: 63.3%
				DOR: 6.6 m
	TNBC	Camrelizumab + apatinib intermittent	10	mOS: 9.5 m
				mPFS: 1.9 m
				1y-OS: 40.0% (12.3–67)
				ORR: 0
				DCR: 40.0%
				DOR: 1.9 m
NCT02401048 Hong et al. (2019)	TNBC/HER2(+)	Durvalumab + ibrutinib	45	mOS: 4.2 m
				mPFS: 1.7 m
				ORR: 3%
NCT02811497 Taylor et al. (2020)	ER(+) HER2(−)	Durvalumab + CC-486 (300mg, q1-14)	28	mPFS: 1.9 m
				mOS: 5.0 m
				DCR: 7.1%
NCT02971761 Yuan et al. (2021)	TNBC	Pembrolizumab + enobosarm	16	mOS: 25.5 m
				mPFS: 2.6 m
				ORR: 13%
				CR: 6%
				DCR: 25% (16w)
NCT02734004 Domchek et al. (2020)	HER2(−)	Olaparib + durvalumab	24	mOS: 21.5 m
				mPFS: 8.2 m
				DCR: 80.0% (at 12w), 50.0% (at 28w)
				ORR: 63.3% (at 12w)
				CR: 3%
NCT02657889 Vinayak et al. (2019)	TNBC	Niraparib + pembrolizumab	47	ORR:18.2%
				CR:9.1%
				DCR:41.8% mPFS: 2.3 m
				6 m-PFS: 28%
				12 m-PFS: 14%
NCT02489448 Ahmed et al. (2020)	TNBC	Nab-paclitaxel + durvalumab- epirubicin + cyclophosphamide + durvalumab	45	pCR: 40%
NCT02622074 Schmid et al. (2020b)	TNBC	Nab-paclitaxel-doxorubicin + cyclophosphamide + pembrolizumab ± carboplatin	60	pCR: ypT0/Tis ypN0 60%, ypT0 ypN0 57%
NCT02685059 Loibl et al. (2019)	TNBC	Durvalumab + NACT (nab-paclitaxel-epirubicin + cyclophosphamide)	88	pCR: 53.4%
	TNBC	Durvalumab + placebo	86	pCR: 44.2%
NCT02819518 Cortes et al. (2020)	TNBC	Pembrolizumab + chemotherapy	566	CPS ≥ 10: mPFS 9.7 m
				CPS ≥ 1: mPFS 7.6 m
				ITT mPFS 7.5 m
	TNBC	Placebo + chemotherapy	281	CPS ≥ 10: mPFS 5.6 m
				CPS ≥ 1: mPFS 5.6 m
				ITT mPFS 5.6 m
NCT03036488 Schmid et al. (2020a)	TNBC	Pembrolizumab + paclitaxel + carboplatin	784	pCR: 64.8%
	TNBC	Placebo + paclitaxel + carboplatin	390	pCR: 51.2%
NCT01042379 Pusztai et al. (2021)	HER2(−)	Placebo + olaparib + paclitaxel-doxorubicin + cyclophosphamide	229	pCR: 20% HER2−, 14% HR+ HER2−, 27% TNBC
	HER2(−)	Durvalumab + olaparib + paclitaxel-doxorubicin + cyclophosphamide	73	pCR: 37% HER2−, 28% HR+ HER2−, 47% TNBC

Abbreviations: PP, per protocol population; pCR, pathological complete response; ORR, objective response rate; DCR, disease control rate; CR, complete response; mPFS, median progression-free survival; mOS, median overall survival; PR, partially response; SD, stable disease; DOR, during of response; NACT, neoadjuvant chemotherapy; CPS, combined positive score; TNBC, triple-negative breast cancer, HER2 human epidermal growth factor receptor-2, HR, hormonal receptor; mBC, metastatic breast cancer; eBC, early-stage breast cancer.

### 4.1 Dual-Drug Therapy

#### 4.4.1 Anti-PD-1/PD-L1 Combined With Chemotherapy

Since the safe and effective dose of ICB and the combination drug in the synergistic regimens were uncertain, phase I/II clinical trials focused on determining the dose applied in the subsequent extensive phase, the safety of the combination regimens, and the indications.

The first report on the efficacy and safety of weekly paclitaxel plus durvalumab in treating mTNBC is an open-label, single-arm, phase I/II clinical trial (NCT02628132) (Ghebeh et al., 2021). During a median follow-up of 24 months, the confirmed ORR was 25% in the intention to treat (ITT) population and 36% (*n* = 5) in per protocol (PP) sets, among whom one patient achieved pathological clinical response (pCR) (Ghebeh et al., 2021). A total of 64% of patients achieved disease control [defined as: complete response (CR) + partially response (PR) + stable disease (SD)], and the median duration of response (DOR, response defined as CR + PR) was 10.0 months (Ghebeh et al., 2021). The mPFS and mOS were 5.0 and 20.7 months, respectively (Ghebeh et al., 2021). Arm C in the clinical trial of FUTURE (NCT03805399) combined the anti-PD-1 drug SH1210 and nab-paclitaxel in heavily pretreated (median of three previous antitumor regimens in the metastatic setting) (mTNBC). A total of 19 patients enrolled were assigned to receive SH1210 plus nab-paclitaxel, among whom 62.5% (*n* = 10) of the PP population and 52.5% of the ITT population reported objective response. Ten patients (62.5%) achieved PR, and the median DOR was 3.1 months (Jiang et al., 2021b). As a novel nontaxane inhibitor, eribulin mesylate was approved a decade ago for metastatic breast cancer (mBC) and has been pretreated with at least two regimens (Mougalian et al., 2021). Eribulin has been reported to exert an antitumor effect by concomitantly inhibiting TGF-β and the PD-1/PD-L1 pathway. Several studies have assessed the effect of eribulin in combination with ICB. In the phase Ib/II trial of ENHANCE I (NCT02513472), mTNBC patients who were treated with eribulin plus pembrolizumab in phase I and 21.8% in phase II achieved ORR. There were remarkably higher ORRs in the PD-L1-positive population, which were 34.5% in phase I and 24.4% in phase II (Tolaney et al., 2021).

Similar, another phase II trial (NCT03051569) compared the efficacy of eribulin plus pembrolizumab with eribulin alone in patients with HR-positive and HER2-negative mBC who had been pretreated (Tolaney et al., 2020). A total of 44 patients with treatment-refractory, pretreated, and HR-positive mBC were included in each group. In contrast to the trial of ENHANCE I, the frequency of ORR in this trial demonstrated no significant difference between the pembrolizumab treatment group and the eribulin treatment group in the PP population (34% versus 27%) or in the PD-L1-positive population (23% versus 45%). No significant difference in PFS between the pembrolizumab arm and the eribulin arm was observed, and the mPFS in the two groups was 4.1 and 4.2 months, respectively (HR = 0.80, 95% CI 0.50–1.26) (Tolaney et al., 2020). In the unmatured survival data,, the mOS was 13.4 months in the pembrolizumab group and 12.5 months in the eribulin-alone group (HR = 0.87, 95% CI 0.48–1.59, *p* = 0.65). In the PD-L1-positive population, the mPFS in the two groups was 4.2 and 4.3 months, and the mOS was 10.4 and 13.1 months, respectively (HR = 1.59, 95% CI 0.50–5.06, *p* = 0.43) (Tolaney et al., 2020). KELLY (NCT03222856) is an another open-label, single-arm, phase II trial that assessed the efficacy of eribulin with or without pembrolizumab (Perez-Garcia et al., 2021). A total of 44 patients with HR-positive, HER2-negative, inoperable, locally recurrent disease or mBC were enrolled, among which 90.9% (*n* = 40) had visceral metastasis (Perez-Garcia et al., 2021). KELLY reported a better ORR of 40.9% (*n* = 18) than that of the NCT03051569 trial, and the clinical benefit (CR + PR + SD) rate was 56.8% (*n* = 25). Among the PD-L1-positive population, the ORR and clinical benefit rate were 38.1% and 42.9%, respectively (Perez-Garcia et al., 2021). During the median follow-up of 12.1 months, the mPFS was 6.0 months, the 1-year OS was 59.1%, and the mOS was not reached (Perez-Garcia et al., 2021). The outcomes derived from KELLY indicated a promising benefit of the combination regimen of pembrolizumab and eribulin.

Then, in phase III clinical trial, a larger patient cohort will be adopted to assess the efficacy of the combinatorial regimen compared with the current standard treatment in a few or only one indications.

The clinical trial IMpassion130 (NCT2425891) is an international, randomized, double-blind, placebo-controlled phase III trial that compared the efficacy of nab-paclitaxel combined with atezolizumab or with placebo in previously untreated metastatic or locally advanced TNBC (Emens et al., 2021). Although significant OS improvement was not observed in PD-L1-unselected patients, those patients with PD-L1-positive expression achieved a substantial increase in OS in IMpassion130. In the latest outcome analysis of the IMpassion130 trial, nab-paclitaxel plus atezolizumab failed to improve OS in patients not screened for the expression status of PD-L1 (Emens et al., 2021). The mOS of the ITT was 21.0 months (95% CI 19.0–23.4 months) in the nab-paclitaxel plus atezolizumab group and 18.7 months (95% CI 16.9–20.8 months) in the nab-paclitaxel plus placebo group (HR = 0.87, 95% CI 0.75–1.02, *p* = 0.077) (Emens et al., 2021). However, a significant improvement in OS was observed in the PD-L1-positive subset of patients; the mOS was 25.4 months (95% CI 19.6–30.7 months) in the nab-paclitaxel plus atezolizumab group and 17.9 months (95% CI 13.6–20.3 months) in the nab-paclitaxel plus placebo group (HR = 0.67, 95% CI 0.53–0.86 (Emens et al., 2021). The 36-month OS of the ITT was 28.1% (95% CI 23.8%–32.4%) in the atezolizumab group and 24.9% (95% CI 20.8%–29.0%) in the placebo group (Emens et al., 2021). Additionally, the 3-year OS rates of PD-L1-positive patients in the two treatment groups were 35.8% (28.8%–42.9%) and 22.2% (15.9%–28.5%), respectively (Emens et al., 2021). The phase Ib trial of GP28328 (NCT01633970) also observed a potential benefit of advanced TNBC patients treated by atezolizumab plus nab-paclitaxel with an ORR of 39.4% (Adams et al., 2019).

Impassion031 (NCT03197935) compared the efficacy of pegylated liposomal doxorubicin and cyclophosphamide plus atezolizumab (atezolizumab plus chemotherapy) or placebo (placebo plus chemotherapy) in early-stage TNBC (Mittendorf et al., 2020). The median follow-up was 20.6 months in the atezolizumab plus chemotherapy group and 19.8 months in the placebo plus chemotherapy group, and pCR was evaluated in 154 patients in each group. Combination therapy with atezolizumab plus chemotherapy was superior to that of placebo plus chemotherapy in early-stage TNBC patients. The pCR rates in the two groups were 58% (*n* = 95) and 41% (*n* = 69), respectively (rate difference 17%, *p* = 0.0044) (Mittendorf et al., 2020). In the PD-L1-positive population, the pCR rates were 69% (*n* = 53) in the atezolizumab plus chemotherapy group and 49% (*n* = 37) in the placebo plus chemotherapy group (rate difference 20%, *p* = 0.021) (Mittendorf et al., 2020). However, the IMpassion031 has not been formally powered for EFS (event-free survival), DFS (disease-free survival), and OS, due to the median survival not being reached at the time of data analysis (Mittendorf et al., 2020). IMpassion131 (NCT03125902) is a double-blind, placebo-controlled, randomized phase III clinical trial that detected the efficacy of paclitaxel with or without atezolizumab for unresectable locally advanced TNBC or mTNBC (Miles et al., 2021). A total of 431 patients were randomized into the atezolizumab plus paclitaxel group, and 220 patients were randomized into the placebo plus paclitaxel group (Miles et al., 2021). Among the 651 randomized patients, 292 were PD-L1-positive, of which 191 received atezolizumab plus paclitaxel treatment and 101 received placebo plus paclitaxel treatment (Miles et al., 2021). The median follow-up of the ITT population was 8.8 months in the atezolizumab plus paclitaxel group and 8.5 months in the placebo plus paclitaxel group. The median follow-up in the PD-L1-positive population was 9.0 months in the atezolizumab plus paclitaxel group and 8.6 months in the placebo plus paclitaxel group (Miles et al., 2021). The proportion of overall response in the ITT population was 54% in the atezolizumab plus paclitaxel group and 47% in the placebo plus paclitaxel group, while in the PD-L1-positive population, it was 63% in the atezolizumab plus paclitaxel group and 55% in the placebo plus paclitaxel group. The latest data showed that the mPFS of the ITT population was 5.9 months in the atezolizumab plus paclitaxel group versus 5.6 months in the placebo plus paclitaxel group (HR = 0.82, 95% CI 0.68–0.98) (Miles et al., 2021). The mOS of the ITT population was 19.2 months in the atezolizumab plus paclitaxel group and 22.8 months in the placebo plus paclitaxel group (HR = 0.82, 95% CI 0.68–0.98). A similar trend toward better efficacy of atezolizumab plus paclitaxel was observed in the PD-L1-positive population. The mPFS was 7.2 months for the atezolizumab plus paclitaxel group and 6.4 months for the placebo plus paclitaxel group (HR = 0.73, 95% CI 0.56–0.96) (Miles et al., 2021). No significant improvement in investigator-assessed PFS was reported in the PD-L1-positive population (HR = 0.82, 95% CI 0.60–1.12, log-rank *p* = 0.20) (Miles et al., 2021). In general, no significant benefit was reported for atezolizumab in combination with paclitaxel in the survival outcomes of mTNBC.

#### 4.1.2 Anti-PD-1/PD-L1 Combined With Irradiation

Numerous preclinical studies confirmed the benefit of combination treatment with radiotherapy and immunotherapy in breast cancer (Cao et al., 2021; Guo et al., 2022); however, no synergistic effects were detected in completed clinical trials of PD-1/PD-L1 blockades in combination with radiotherapy.

In a phase II one-arm trial (NCT03366844), patients with HR-positive, HER2-negative mBC were treated with palliative irradiation (4 Gy × 5) concurrent with pembrolizumab. Among all eight patients enrolled, no objective response was observed. Two of the eight patients experienced SD less than 6 weeks, and five experienced PD. The mPFS and mOS were 1.4 and 2.9 months, respectively (Barroso-Sousa et al., 2020b). TONIC (NCT02499367) detected the effect of immune induction strategies, including radiotherapy, in enhancing the sensitivity of PD-1 blockade of nivolumab in mTNBC. A total of 13 patients received 8 Gy × 3 irradiation combined with nivolumab, and 8% of patients achieved objective response, including one who achieved PR (Voorwerk et al., 2019). The efficacy and safety of combination treatment with anti-PD-1/PD-L1 agents and radiotherapy remain uncertain and should be further analyzed. Promisingly, ongoing trials will offer a comprehensive perspective on combining PD-1/PD-L1 blockade and radiotherapy for the treatment of breast cancer.

#### 4.1.3 Anti-PD-1/PD-L1 Combined With Anti-HER2 Therapy

The phase Ib study GO29381 (NCT02605915) explored the effect of atezolizumab in combination with trastuzumab/pertuzumab (A + T + P), atezolizumab with an antibody–drug conjugate of ado-trastuzumab (T-DM1) (A + T-DM1), or atezolizumab with trastuzumab/pertuzumab and docetaxel (A + T + P + D) in unresectable HER2-positive breast cancer and the effect of atezolizumab with trastuzumab/pertuzumab (A + T + P) or with T-DM1 (A + T-DM1) in neoadjuvant therapy for early HER2-positive breast cancer (eBC) (Hamilton et al., 2021). Patients with mBC in the A + T + P treatment group achieved a 14% ORR, those in the A + T-DM1 treatment group achieved an ORR of 35%, and mBC patients treated with A + T + P + D achieved an ORR of 100%. A total of 65% of patients with eBC who received A + T + P and 70% who received A + T-DM1 achieved pCR (Hamilton et al., 2021). Remarkable results have been achieved in the maintenance treatment of HER2-positive breast cancer with anti-HER2 therapy combined with PD-1/PD-L1 blockades. The phase Ib clinical trial of CCTG IND.229 (NCT02649686) explored the efficacy of durvalumab in combination with trastuzumab in HER2-positive mBC (Chia et al., 2019). Among the 14 subjects who were evaluable for response, 29% (*n* = 4) demonstrated stable disease, while no response was observed. The median response duration was 2.7 months, and the mPFS was 1.35 months (95% confidence interval CI, 1.25–1.71 months) (Chia et al., 2019). The estimated 6-month OS was 51.6%, and the 1-year OS was 17.2%; however, the estimated 6-month PFS was 0% (Chia et al., 2019). PANACEA (NCT02129556) is a single-arm, phase Ib/II clinical trial that assessed the efficacy of pembrolizumab plus trastuzumab in trastuzumab-resistant, advanced, HER2-positive breast cancer (Abraham and Weiss, 2004). Six patients were enrolled in phase Ib, among whom three were assigned to the 2 mg/kg pembrolizumab group and three were assigned to the 10 mg/kg pembrolizumab group. Then, 52 patients were enrolled in phase II (Abraham and Weiss, 2004). The median follow-up was 25.7 months, during which an objective response rate of 17% and a disease control rate of 17% were confirmed in the phase Ib, 2 mg/kg pembrolizumab group (Abraham and Weiss, 2004). In phase II, the median follow-up for the PD-L1-positive population was 13.6 months. A total of 15% (*n* = 6) of patients had a centrally confirmed objective response, among which one patient (3%) achieved CR. The mPFS of this group of patients was 2.7 months, the estimated 6-month PFS was 25%, and the 12-month PFS was 12% (Abraham and Weiss, 2004). The mOS was not reached, and the estimated 6-month OS and 12-month OS were 87% and 65%, respectively (Abraham and Weiss, 2004). In the *post hoc* combined analysis of the Ib and II PD-L1-positive population (*n* = 46), the objective response rate was 15%, with another 8% (*n* = 4) of patients experiencing stable disease. The mPFS was 2.7 months, and the 6-month and 12-month PFS rates were 24% and 13%, respectively (Abraham and Weiss, 2004). The phase II double-blind trial of KATE2 (NCT02924883) randomly assigned patients to receive plus T-DM1 plus atezolizumab (*n* = 133) or placebo (*n* = 69) treatment (Emens et al., 2020). A total of 45% (*n* = 60) of patients treated with atezolizumab achieved an objective response, among which eight patients achieved CR, and 43% (*n* = 30) of patients in the placebo group reported an objective response, which included five patients who achieved CR (OR = 1.07, 95% CI 0.60–1.91) (Emens et al., 2020). In the *post hoc* analysis, the ORR in the PD-L1-positive population was 54% (*n* = 30) in the atezolizumab group and 33% (*n* = 9) in the placebo group. The median follow-up of the two groups was 8.5 and 8.4 months, during which 8.2 months of PFS and 6.8 months of PFS were observed, respectively (HR = 0.82, 95% CI 0.55–1.23, *p* = 0.33). In the PD-L1-positive population, the mPFS was 8.5 months in the atezolizumab group and 4.1 months in the placebo group (HR = 0.6, 95% CI 0.32–1.11, *p* = 0.099). The mOS was not reached, and the stratified HR was reported to be 0.74 (95% CI 0.42–1.30) (Emens et al., 2020). In the interim analysis, the 12-month OS was 89% (95% CI 84%–94%) in the atezolizumab group and 89% (95% CI 81%–98%) in the placebo group (Emens et al., 2020).

#### 4.1.4 Anti-PD-1/PD-L1 Combined With Other Types of Targeted Therapy

Preclinical research had observed an attractive success of immune check point antagonist in combination with antiangiogenics. Thus, an attempt at this combinatorial strategy has been made in clinical trials including antiangiogenics antibody and tyrosine kinase inhibitor (TKI). Patients treated by durvalumab plus bevacizumab demonstrated encouraging increase in survival outcomes compared to the durvalumab monotherapy group in the trial of SAFIRO2-BREAST IMMUNO, in which the mPFS and mOS was 2.7 and 21.7 months, respectively (Bachelot et al., 2021). Besides, a trend of longer OS was observed in the HR-positive subset compared to the triple-negative subset (mOS 19.8 months vs. 7.4 months, *p* = 0.11) (Quintela-Fandino et al., 2020). Among the 25 patients who received treatment, clinical benefit (CR + PR + SD) was observed in 60% (*n* = 15) of patients at 8 weeks and increased to 44% at 16 weeks. The mPFS and mOS were 3.5 and 11 months, respectively (Quintela-Fandino et al., 2020). Apatinib belongs to the TKI family and targets vascular endothelial growth factor receptor (VEGFR) to generate antiangiogenic signals. Another phase II trial (NCT03394287) reported the efficacy of camrelizumab combined with apatinib in advanced TNBC (Liu et al., 2020). The ORR was 43.3% in patients who received continuous dosing, while no object response was observed in the intermittent dosing group. The disease control rate (CR + PR + SD) was 63.3% in the continuous dosing group and 40% in the intermittent dosing group, and the mPFS in the two groups was 3.7 and 1.9 months, respectively (Liu et al., 2020).

In a one-arm clinical trial (NCT02401048) that combined BTK inhibitor and durvalumab, the mPFS of breast cancer patients was 1.7 months, and the mOS was 4.2 months (Hong et al., 2019).

Other kinds of targeted therapies have also been developed in combination with ICB. Study NCT02811497 explored the efficacy of the DNA HMA CC-486 in enhancing the immunotherapy response of tumors to ICB (Taylor et al., 2020). Twenty-eight patients were allocated to receive regimen A: CC-486 (300 mg, d1–d14) plus durvalumab (*n* = 19) and regimen B: CC-486 (100 mg, d1–d21), vitamin C plus durvalumab (*n* = 9). This study reported a disease control rate of 7.1%, a mPFS of 1.9 months, and a mOS of 5.0 months, which failed to achieve robust pharmacodynamic and clinical activity in breast cancer (Taylor et al., 2020). Luminal AR-positive is a subtype of TNBC characterized by the expression of AR. Targeting AR is theoretically effective in this type of TNBC; however, the effect of a single anti-AR drug is limited (Gucalp et al., 2013). Olaparib is a kind of PARPi that showed a definite effect in breast cancer patients with germline variants of *BRCA1/2* by inhibiting the impairment of DNA damage and leading to synthetic lethality. Previous research found an interaction between DNA damage caused by PARPi and the immune system and the upregulation of PD-L1 expression induced by PARPi, providing evidence for combining PARPi and ICB. Substantial preclinical evidence justified the combination of PARPi and ICB, and effort has been made to develop dual-drug therapy into the clinical practice of breast cancer. The trial of NCT02971761 explored the regimen of enobosarm (GTx-024) synergized with pembrolizumab in AR-positive mTNBC, which reported 6% (*n* = 1) CR, 6% PR (*n* = 1), and 13% SD (*n* = 2). The median follow-up was 24.9 months, and the mPFS and mOS were 2.6 and 25.5 months, respectively. The combination of enobosarm and pembrolizumab demonstrated good tolerance and safety and a modest clinical response (Yuan et al., 2021). MEDIOLA is an open-label, phase I/II trial (NCT02734004) to assess the response of *BRCA*-mutated mBC to the regimen of PARPi plus durvalumab (Domchek et al., 2020). Twenty-four of the 30 patients (80%) who received treatment had disease control at 12 weeks, and 50% (*n* = 15) of the patients had disease control at week 28. During the 6.7-month follow-up, the mPFS was 8.2 months, and at a median follow-up of 19.8 months, the mOS was 21.5 months (Domchek et al., 2020). In the TNBC population, the mPFS was 4.9 months, and the mOS was 20.5 months. Similar clinical outcomes were observed in patients with both BRCA1 and BRCA2 mutations. The MEDIOLA trial did not demonstrate benefit in the combination regimen compared to previous research concerning the monotherapy of PARPi (Domchek et al., 2020).

### 4.2 Multidrug Therapy

To date, most multidrug regimens in clinical trials have been ICB combined with chemotherapeutic drugs, indicating that the exploration of the combinatorial strategy of ICB is at its early phase. The phase I/II trial (**NCT02489448**) explored the efficacy of durvalumab concurrent with nab-paclitaxel followed by doxorubicin and cyclophosphamide in early-stage TNBC at the neoadjuvant phase (Ahmed et al., 2020). pCR was achieved in 71% of patients with PD-L1-positive tumors after surgery (Ahmed et al., 2020). Phase Ib KEYNOTE-173 (**NCT02622074**) explored the efficacy of pembrolizumab combined with neoadjuvant chemotherapy in high-risk, early-stage TNBC. Pembrolizumab plus a total of six different doses of chemotherapy regimens demonstrated a 60% ORR and confirmed that pCR was correlated with tumor PD-1 expression and stromal tumor infiltrating lymphocyte (sTIL) levels (Schmid et al., 2020b). The randomized phase II trial of GeparNuevo (**NCT02685059**) compared the efficacy of durvalumab in combination with anthracycline taxane-based neoadjuvant therapy in early TNBC (Loibl et al., 2019). A total of 174 patients were randomized, of which 88 patients were assigned to receive the durvalumab treatment group and 86 were assigned to the placebo treatment group. A total of 53.4% (*n* = 47) of patients treated with durvalumab achieved pCR, and 44.2% (*n* = 38) of patients treated with placebo achieved pCR (OR = 1.45, 95% CI 0.80–2.63, *p* = 0.224). Among the PD-L1-positive population, 58.0% in the durvalumab treatment group and 44.4% in the placebo treatment group achieved pCR (*p* = 0.445) (Loibl et al., 2019). KRYNOTE-355 (**NCT02819518**) compared the efficacy of chemotherapy plus pembrolizumab versus chemotherapy plus placebo in previously untreated locally recurrent unresectable or mTNBC (Cortes et al., 2020). In this phase III double-blind trial, 847 patients were randomly assigned 2:1 into the pembrolizumab plus chemotherapy group (*n* = 566) and placebo plus chemotherapy group (*n* = 281). The median follow-up period was 25.9 months in the pembrolizumab plus chemotherapy group and 26.3 months in the placebo plus chemotherapy group (Cortes et al., 2020). The PD-L1 expression status was detected before treatment and indicated by the combined positive score (CPS), which was defined as the number of PD-L1-positive cells (Cortes et al., 2020). Among patients with CPS ≥ 10, 220 and 103 patients were assigned to the pembrolizumab plus chemotherapy group and the placebo plus chemotherapy group, respectively, and 425 and 211 of those with CPS ≥ 1 were assigned to the pembrolizumab plus chemotherapy group and the placebo plus chemotherapy group, respectively (Cortes et al., 2020). Among patients with CPS ≥ 10, the mPFS was 9.7 months in patients treated with pembrolizumab plus chemotherapy, and it was 5.6 months in patients who received placebo plus chemotherapy (HR = 0.65, 95% CI 0.49–0.86), while the mPFS was 7.6 and 5.6 months in the two groups, respectively, among patients with CPS ≥ 1 and 6.3 months and 6.2 months in patients with CPS < 1 (Cortes et al., 2020). In the ITT population, the mPFS was 7.6 months in the pembrolizumab plus chemotherapy treatment group and 5.6 months in the placebo plus chemotherapy treatment group (Cortes et al., 2020). KEYNOTE-522 (**NCT03036488**) adopted previously untreated stage II-III TNBC to compare the effect of the neoadjuvant regimen of paclitaxel and carboplatin combined with pembrolizumab (*n* = 784) or placebo (*n* = 390). A total of 64.8% of patients in the pembrolizumab group and 51.2% of patients in the placebo group achieved pCR (*p* < 0.001). After a median follow-up of 15.5 months, 7.4% (*n* = 58) and 11.8% (*n* = 46) of patients had disease progression in the two groups, respectively (Schmid et al., 2020a).

We retrieved only one trial of a multidrug regimen involving the PARPi olaparib. The I-SPY2 trial (**NCT01042379**) assessed the efficacy of durvalumab plus olaparib and paclitaxel in high-risk HER2-negative stage II-III breast cancer (Pusztai et al., 2021). In phase II/III I-SPY2, researchers evaluated the benefit of the combination regimen of durvalumab, olaparib, and weekly paclitaxel in neoadjuvant treatment for early breast cancer (Pusztai et al., 2021). Seventy-three patients were allocated to receive the combination therapy, and 299 were randomized to the paclitaxel control arm. The estimated pCR of TNBC in the combination therapy group was 47%, and it was 27% in the control arm (Pusztai et al., 2021). The phase II I-SPY2 trial reported that the pCR rate of TNBC improved from 22 to 60% when pembrolizumab was added to neoadjuvant chemotherapy (Nanda et al., 2020). Meanwhile, in a phase III trial, the addition of pembrolizumab to chemotherapy further boosted the pCR rate from 51.2% to 64.8% (Schmid et al., 2020a).

## 5 Adverse Events of the Combination Therapeutic Strategy With PD-1/PD-L1 Blockade

Although the incidence is low and milder than that induced by other cancer therapies, adverse events caused by ICBs are characterized by a unique profile of inflammatory features, such as pneumonitis, hepatitis, and colitis, which was named immune-related AEs (irAEs), often need special management (Kennedy and Salama, 2020; Zhou et al., 2021b). The incidence of severe irAEs (≥ grade 3) had been reported of 20%–30% in patients received ipilimumab, and 10%–15% in those treated with anti-PD-1 agents (Martins et al., 2019; Darnell et al., 2020).

The trial (**NCT02628132**) that first detected the efficacy of paclitaxel concurrent with durvalumab in mTNBC reported a frequency of 71% of AEs, among which headache (29%) and peripheral neuropathy (21%) were the two most common. A total of 21% (*n* = 3) of patients experienced grade 3/4 AEs (Ghebeh et al., 2021). In the umbrella trial of FUTURE (**NCT03805399**), group C explored the clinical efficacy and safety of anti-PD-1 (SH1210) in combination with nab-paclitaxel. The top three most common AEs reported in the FUTURE were anemia (67%), leukopenia (59%), and neutropenia (43%), but the overall AEs incidence and severity were moderate and acceptable. ENHANCE 1 (**NCT02513472**) reported a rate of 43% pembrolizumab-related AEs, among which 12% were grade 3-4 (Tolaney et al., 2021). A clinical trial assessed the efficacy of eribulin in combination with pembrolizumab (**NCT03051659**) and reported that AEs occurred in 100% of patients in both arms, and the frequency of grade 3 or higher AEs was 68% in the pembrolizumab arm and 61% in the eribulin arm (Tolaney et al., 2020). The most common grade 3 or higher AEs were neutropenia (37% in both arms), febrile neutropenia (9% versus 14%), and liver enzyme elevation (14% versus 7%) (Tolaney et al., 2020). The incidence of all-cause treatment-emergent AEs (TEAEs) was 100% in the KELLY trial (**NCT03222856**), and SAEs occurred in 31.8% (*n* = 14) of patients (Perez-Garcia et al., 2021), among which febrile neutropenia (6.8%) and neutropenia (5.0%) were the most common. One patient suffered from a fatal TEAE due to cardiac arrest unrelated to the study treatment (Perez-Garcia et al., 2021). Impassion130 (**NCT02425891**), the most frequent AEs in both the atezolizumab arm and the placebo arm were alopecia, fatigue, and nausea (Emens et al., 2021). Fifty-one percent (*n* = 233) of patients in the atezolizumab group and 43% (*n* = 183) of patients in the placebo group were reported as having grade 3-4 AEs, and the difference in AEs frequencies between the two groups was not statistically significant (Emens et al., 2021). A total of 19% (*n* = 88) of patients in the atezolizumab group and 8% (*n* = 36) in the placebo group experienced treatment discontinuation, which was mostly due to neuropathy (Emens et al., 2021). In the IMpassion031 (**NCT03197935**), the frequencies of all-cause any-grade AEs were 99% (*n* = 163) in the atezolizumab plus chemotherapy group and 100% (*n* = 167) in the placebo plus chemotherapy group. Sixty-three percent (*n* = 103) of patients in the atezolizumab plus chemotherapy group and 60% (*n* = 101) in the placebo plus chemotherapy group experienced grade 3-4 AEs (Mittendorf et al., 2020). Serious adverse events (SAEs) were reported in 30% of patients (*n* = 50) in the atezolizumab plus chemotherapy group and 18% (*n* = 30) in the placebo plus chemotherapy group. Impassion131 (**NCT03125902**) reported a relatively high SAEs incidence (25% vs. 18%) and AEs leading to treatment discontinuation (21% vs. 15%) in the atezolizumab plus paclitaxel group than the placebo control group. Besides, the special interest AEs were also more frequent in the atezolizumab treating group than the placebo group (Miles et al., 2021). The incidence of AEs was higher in the phase Ib trial of **NCT01633970** when compared to Impassion 131 (Adams et al., 2019). A total of 100% incidence of all-grade of AEs and 73% of grade 3/4 Treatment-related AEs (TRAEs) were observed in this trial. Additionally, the most common grade 3/4 AEs attributed to atezolizumab were diarrhea (6%) and colitis (3%), and there 9% (*n* = 3) patients discontinued atezolizumab treatment due to TRAEs (Adams et al., 2019).

Pembrolizumab plus irradiation (**NCT03366844**) reported an 87.5% of all-cause of AEs rates, in which the most common were fatigue (50%), increased aspartate aminotransferase (50%), anemia (25%), arthralgia (25%), dyspnea (25%), and nausea (25%). Grade 3 AEs were reported in only one patient (12.5%) with increased aspartate aminotransferase levels (Barroso-Sousa et al., 2020b). The TONIC trial (**NCT02499367**) reported an obviously lower any-grade TRAEs incidence of 28%, but the grade 3–5 irAE incidence was slightly higher (19%) in this study (Voorwerk et al., 2019).

In the trial of GO29381 (**NCT02605915**), 90% of patients who received atezolizumab plus T-DM1 suffered from irAEs. In addition, 85.2% of patients received atezolizumab combined with trastuzumab and pertuzumab, and 83.3% of patients who received atezolizumab combined with trastuzumab, pertuzumab and docetaxel suffered from irAEs (Hamilton et al., 2021). In the CCTG IND. 229 (**NCT02649686**), the most commonly reported AEs were fatigue, nausea, constipation, headache, etc. Two subjects reported grade 3 lymphocytopenia, one patient experienced grade 3 amylase elevation, and two patients suffered grade 4 hyperglycemia (Chia et al., 2019). In phase Ib of PANACEA (**NCT02129556**), no dose-limiting toxicities, cardiovascular toxic effects, or deaths were reported (Loi et al., 2019). However, 97% (*n* = 56) experienced AEs, including 71% (*n* = 41) TEAEs, of whom 29% (*n* = 17) were grade 3 or higher. The most common grade 3 or higher AEs in the trial of KATE2 (**NCT02924883**) were thrombocytopenia (13% in the atezolizumab versus 4% in the placebo group), increased aspartate aminotransferase (8% versus 3%), anemia (5% versus 0), neutropenia (5% versus 4%), and increased alanine aminotransferase (5% versus 3%) (Emens et al., 2020). SAEs occurred in 33% (*n* = 43) of patients treated with atezolizumab and 19% (*n* = 13) of patients treated with placebo. TEAEs were reported in 19% (*n* = 25) of patients in the atezolizumab group and 3% (*n* = 2) in the placebo group, and the most common was pyrexia leading to hospitalization, which occurred in seven patients.

The scope of AEs result from the combinatorial strategy of ICB with antiangiogenesis was similar to the common antitumor regimens in breast cancer. The most common AEs of all-grade in **NCT02802098** was asthenia with an incidence of 20%. In term of the camrelizumab plus apatinib regimen (**NCT03394287**), the most common AEs were increased aspartate aminotransferase and alanine aminotransferase levels. Grade 3 or worse AEs were observed in 26.7% of patients in the continuous dosing cohort and 20% of patients in the intermittent dosing cohort, respectively (Liu et al., 2020). In a clinical trial combining the BTK inhibitor ibrutinib and durvalumab (**NCT02401048),** any grade of TEAEs was observed in all breast cancer patients, and the incidence of grade 3 or worse TEAEs was 78% (Hong et al., 2019). Study **NCT02811497** reported that grade 3 or higher AEs (neutropenia) occurred in 18% of patients. One patient in the low-dose cohort experienced a grade 3 AST/ALT increase, and another patient experienced grade 3 anemia. In the high-dose cohort, two patients experienced grade 3 hyponatremia, but no patients required dose reduction of CC-486 (Taylor et al., 2020). The trial detected the effect of the nonsteroidal selective androgen receptor modulator (SARM) plus pembrolizumab (**NCT02971761**) and reported good tolerance, in which no grade 4 or worse AEs was observed and the incidence of musculoskeletal ache, dry skin, and diarrhea was 6% (Yuan et al., 2021). The clinical trial MEDIOLA (**NCT02734004**) reported that 32% (*n* = 11) of patients experienced grade 3 or worse AEs, and the most common AEs were anemia (12%), neutropenia (9%), and pancreatitis (6%) (Domchek et al., 2020). In the TOPACIO (**NCT02657889**) study, there were 93% patients suffered from any grade of TRAEs, among which 58% were grade 3 of higher, indicating a remarkable TRAEs frequency induced by the regimen of PARPi combining pembrolizumab.

**FIGURE 4:**
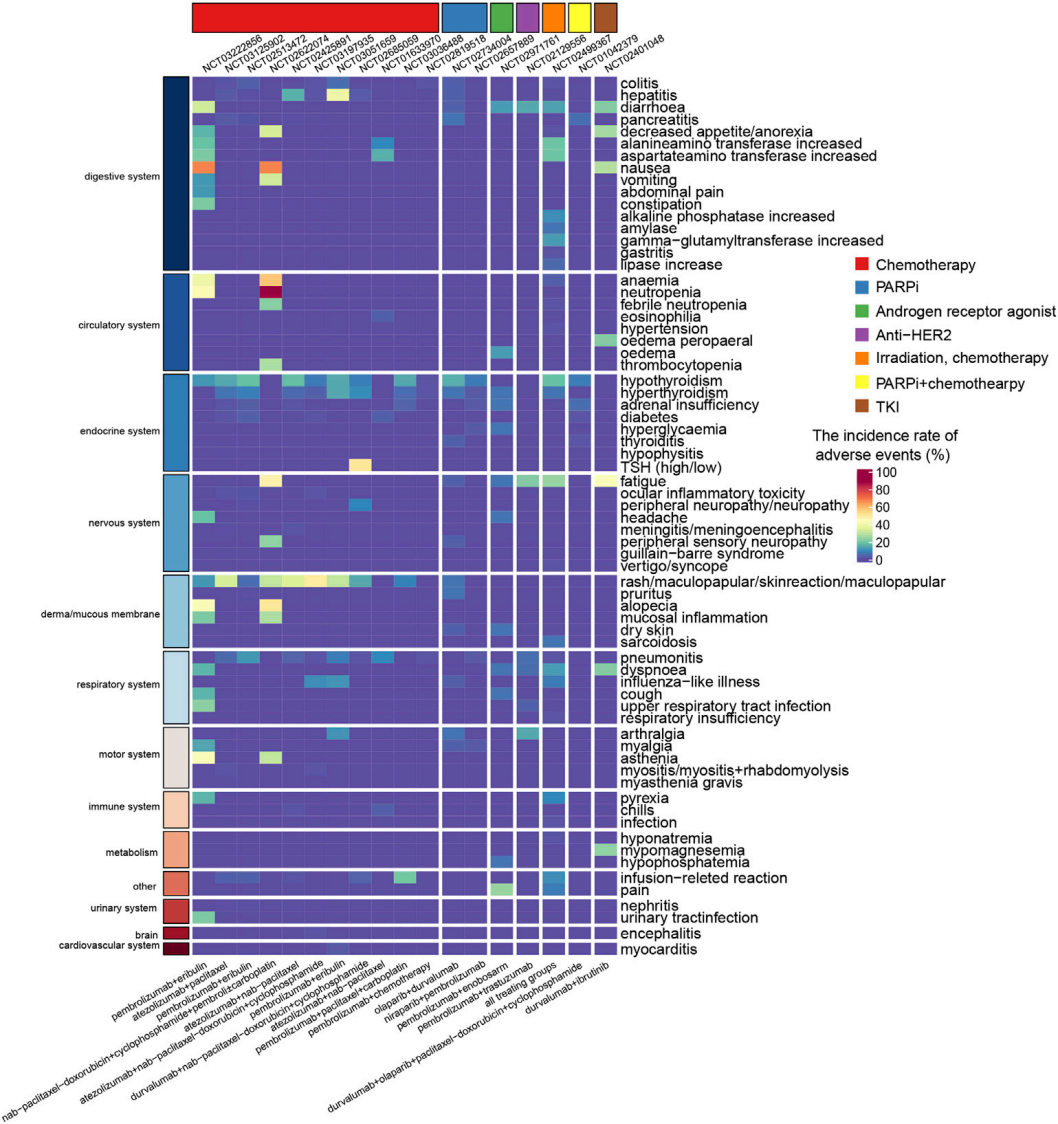
Heatmap showing the incidence rate of immune-related adverse events induced by combination therapy with PD-1/PD-L1 blockade. The color gradient shows the incidence rate of adverse events, where red and blue colors indicate a high and low rate. The regimen of nab-paclitaxel-doxorubicin+cyclophosphamide was nab-paclitaxel followed by doxorubicin and cyclophosphamide.

In the multidrug combinatorial strategies, a relatively higher AEs incidence compared to those dual-drug regimens. The most common dose-limited toxicity in all the six therapy groups of KEYNOTE-173 (**NCT02622074**) was febrile neutropenia, which occurred in ten patients across all groups (Schmid et al., 2020b). Regimens included paclitaxel demonstrated severer toxicity than nab-paclitaxel, and high toxicity incidence had been observed when carboplatin was combined. TRAEs were discussed separately in KEYNOTE-173. There were 88% patients suffered from neutropenia, 67% patients suffered from nausea, and 57% patients occurred anemia, all of which were defined as TRAEs (Schmid et al., 2020b). Ninety percent of TRAEs were grade 3 or severer and 40% patients occurred serious TRAEs and 18% patients discontinued pembrolizumab due to TRAEs. There were 30% AEs were presumed induced by immunologic mechanism (Schmid et al., 2020b). The combination regimen of durvalumab with nab-paclitaxel followed by dose-dense epirubicin and cyclophosphamide in the GeparNuevo (**NCT02685059**) trial reported an AEs incidence of 22.7% (*n* = 20) in the ICB combining group, and 19.8% in the placebo group (Loibl et al., 2019). There were five patients who discontinued durvalumab due to irAEs and less frequent AEs were reported in durvalumab treating group than the placebo group (Loibl et al., 2019). KEYNOTE-355 (**NCT02819518**) reported 98% and 95% AEs rates in the pembrolizumab combined with chemotherapy group and the placebo combined with chemotherapy group, respectively (Cortes et al., 2020). The frequencies of grade 3 or higher treatment-related AEs were 68% and 67% in the pembrolizumab-containing group and the placebo group, respectively. The I-SPY2 trial (**NCT01042379**) reported a 56% (*n* = 41) incidence of grade 3 or worse AEs in the combination treatment arm and 34% (*n* = 102) in the control arm. This study also reported that 27.4% of patients (*n* = 20) experienced immune-related AEs in the durvalumab-containing arm, while the incidence was only 2% (*n* = 6) in the control arm (Pusztai et al., 2021). The results of these clinical trials provide a clinical reference for the immune-related toxicity profiles of PD-1/PD-L1 blockade-based combination therapies (Figure 4). The profiles of AEs (Supplementary Figure S1) and SAEs (Supplementary Figure S2) were visualized grouped by the combinatorial strategies and the organs/systems that occurred the event.

## 6 Predictive Biomarkers for PD-1/PD-L1 Blockade in Combination With Other Cancer Treatment Regimens

Similar to conventional anticancer treatments, the application of PD-1/PD-L1 blockade alone or in combination with other treatment regimens in cancer therapy is also curtailed by a low response rate in certain cancers, immune-related toxicity, and innate and acquired drug resistance. Thus, identifying optimal biomarkers for discriminating cancer patients who are responsive to PD-1/PD-L1 blockades alone or in combination with other approaches and accurately monitoring therapeutic efficacy are of great clinical importance. The following sections outline biomarkers for predicting the response to combinatorial therapy of PD-1/PD-L1 blockade in patients with breast cancer.

### 6.1 PD-L1 Expression

Direct assessment of PD-L1 expression has been widely used to predict treatment response to anti-PD-1 or anti-PD-L1 therapies. Regarding the predictive value of PD-L1 expression for the response to combination therapy, emerging evidence demonstrates that PD-L1 expression has clinical use in breast cancer patient stratification. One clinical trial investigating the safety and efficacy of a PD-1 blockade (pembrolizumab) in combination with trastuzumab in HER2-positive breast cancer documented no responses in the PD-L1-negative cohort, whereas PD-L1-positive patients achieved a 15% response rate (90% CI, 7%–29%) (Loi et al., 2019). The results from the clinical trial GO29381 showed that PD-L1 in immune cells was the only biomarker that increased on atezolizumab/T-DM1 exposure in HER2-positive breast cancer (Hamilton et al., 2021). In the phase Ib trial of durvalumab in combination with trastuzumab, no significant clinical activity was observed in patients with heavily pretreated HER2-positive PD-L1-negative mBC (Chia et al., 2019), further supporting the importance of PD-L1 as a selection biomarker for PD-1/PD-L1 blockade in combination with other therapies (Table 3).

**TABLE 3:** Predictive biomarkers for PD-1/PD-L1 blockade in combination with other cancer treatment regimens.

Biomarker	Additional therapy	Association with favourable clinical outcome	Tissue type for biomarker assessment	Assay type for biomarker assessment	Clinical trials
PD-L1 expression	Chemotherapy	Positive	Tumor	QIF	NCT02489448
	Chemotherapy	Positive	Tumor	IHC	NCT02622074
	Chemotherapy	Positive	Tumor	IHC	NCT02685059
	Anti-HER2	Positive	Tumor	IHC	NCT02605915
	Anti-HER2	Positive	Tumor	IHC	NCT02649686
	Anti-HER2	Positive	Tumor	IHC	NCT02924883
	PARPi	Positive	Tumor	IHC	NCT02734004
TIL status	Chemotherapy	Positive	Tumor	HE	NCT02622074
	Chemotherapy	Positive	Tumor	HE	NCT02685059
	Anti-HER2	positive	Tumor	HE	NCT02924883
	Anti-HER2	Positive	Tumor	HE	NCT02129556
	TKI	Positive	Tumor	HE	NCT03394287
	PARPi	Positive	Tumor	—	NCT02734004
Immune signatures	Irradiation, chemotherapy	Positive	Tumor	NanoString	NCT02499367
	Anti-HER2	Positive	Tumor	RNA-seq	NCT02924883
	PARPi + chemotherapy	Positive/Negative	Tumor	Microarray	NCT01042379
Specific T cell subtypes-Tregs	Anti-angiogenesis	Negative	Blood	Gallios cytometer	NCT02802098
TMB	Androgen receptor agonist	Positive	Tumor	TEMPUS xT genome alteration panel	NCT02971761

Abbreviations: TIL, tumor infiltrating lymphocyte; QIF, quantitative immunofluorescence; IHC, immunohistochemistry; HE, hematoxylin and eosin stain; TMB, tumor mutation burden.

The GeparNuevo trial systematically evaluated the potential of indicators related to PD-L1 expression as biomarkers to predict response to the combination therapy of durvalumab and nab-paclitaxel. PD-L1-tumor cells were response predictor in the durvalumab arm (*p* = 0.045), whereas PD-L1-immune cells were more suitable as a predictive indicator in the placebo arm (*p* = 0.040) (Loibl et al., 2019). The predictive potential of tumor cells with PD-L1 expression was also confirmed in the phase Ib KEYNOTE-173 study, in which higher PD-L1 expression was positively associated with pCR and response rates to neoadjuvant pembrolizumab plus chemotherapy (Schmid et al., 2020b). Conversely, in the phase III IMpassion130 study, a clinically meaningful OS benefit was documented only in the PD-L1-immune cell cohort (Emens et al., 2021). Additionally, PD-L1 expression in CD68+ cells was also associated with higher rates of pCR to durvalumab and chemotherapy in TNBC (Ahmed et al., 2020). However, the predictive potential of PD-L1 expression was not seen in a randomized clinical trial of patients with HR-positive, ERBB2-negative mBC (Tolaney et al., 2020). Biomarker analysis indicated that PD-L1 status, TILs, tumor mutation burden (TMB), and genomic alterations were not associated with PFS in the pembrolizumab plus eribulin arm (Tolaney et al., 2020; Bai et al., 2021) (Table 3).

PD-L1 expression has been suggested as a potential predictive biomarker to identify patients who are the most likely to benefit from PD-1/PD-L1 blockade combination approaches in breast cancer. However, the predictive value of PD-L1 expression is still controversial, and contradictory results have been reported as discussed above. Further efforts to explore the predictive value of PD-L1 expression in predicting the clinical efficacy of PD-1/PD-L1 blockade alone or in combination are needed.

### 6.2 TIL Status

Tumor-infiltrating lymphocytes have become an invaluable treatment stratification marker in anti-PD-1/PD-L1 monotherapy as they are representative of the TME. The application potential of TILs in the setting of combination therapy with PD-1/PD-L1 blockades has gradually emerged. The baseline TILs from a metastatic lesion may be a promising biomarker for enhanced clinical activity, which can identify HER2-positive breast cancer patients with a higher chance of responding to pembrolizumab and trastuzumab (Loi et al., 2019). Similarly, a high percentage of baseline stromal TILs was associated with more favorable outcomes with the combinational treatment of camrelizumab and apatinib in TNBC (Liu et al., 2020). In GeparNuevo, only stromal TILs but not intratumoural TILs (iTILs) before therapy predicted a higher pCR rate in both arms, while altered dynamics of iTILs between baseline and postwindow phage were specifically predictive of pCR in patients treated with durvalumab plus nab-paclitaxel followed by standard epirubicin plus cyclophosphamide (Loibl et al., 2019). These findings support the value of TIL status as a potential predictive biomarker of clinical benefit from combinational therapy with PD-1/PD/L1 blockade in breast cancer (Table 3).

Note that TIL status appeared to be highly correlated with PD-L1 expression in breast cancer (Denkert et al., 2015). Exploratory analysis of the MEDIOLA phase II trial showed a modest increase in benefit from combination therapy in patients with PD-L1 positivity and tumors with higher stromal CD8+ TILs (Domchek et al., 2020), in concordance with other studies (Chia et al., 2019; Loibl et al., 2019; Schmid et al., 2020b). Therefore, selection for patients with PD-L1 positivity and high TIL levels in future studies of breast cancer testing anti-PD-1/PD-L1 drugs combined with other therapy approaches seems warranted.

### 6.3 Emerging Biomarkers

In addition to PD-L1 and TILs, emerging biomarkers to predict responses have been extensively studied for combination therapy. A study assessed the baseline and dynamic changes of tumor and blood biomarkers to predict the clinical response to a combinational therapy of ICB and anti-angiogenesis in patients with advanced TNBC (Liu et al., 2021). The results demonstrated that indicators related to cytokines, chemokines, growth factors, checkpoint-related proteins and blood immune cell subpopulations may allow for improved patient selection for camrelizumab plus apatinib combinational therapy (Liu et al., 2021). In a pilot clinical trial examining the efficacy of combining durvalumab and bevacizumab for advanced HER2-negative breast cancer, the predictive roles of specific T cell subtypes for the clinical response to combination therapy were highlighted, pointing toward Tregs as a potential biomarker (Quintela-Fandino et al., 2020). Breast cancer has a low TMB compared with other immunogenic cancers, while TNBC has a relatively higher TMB than other subtypes of breast cancer (Yarchoan et al., 2017; Barroso-Sousa et al., 2020a). A recent analysis of patients with TNBC treated with pembrolizumab and enobosarm found that the patient with CR had the highest TMB (Yuan et al., 2021) (Table 3).

Gene expression profiling typically provides an estimation of the abundances and functional status of distinct cell types in the TME, particularly immune infiltration, which offers a more nuanced detection of an immune-activated state. Therefore, gene expression signatures can be employed as surrogates for the assessment of tumor response to combination therapy. After doxorubicin and cisplatin induction, immune-related genes involved in the PD-1/PD-L1 axis and T cell cytotoxicity pathways were upregulated in TNBC, which established a favorable TME and enhanced the likelihood of response to PD-1 blockade (Voorwerk et al., 2019). The T effector cell gene signature, CD8 immunohistochemistry expression, and TILs also seemed to be associated with PFS (Emens et al., 2020). The predictive functions of seven immune signatures were assessed in the I-SPY2 trial, corresponding to various immune cell types, STAT1 cytokine signaling, and macrophage/T cell ratios (Pusztai et al., 2021). Immune signatures were positively associated with pCR in the durvalumab/olaparib arm, underscoring the predictive potential of immune signatures (Pusztai et al., 2021). Clinical trials are needed to evaluate whether additional biomarkers can improve patient selection for combination therapy of ICB with other treatments (Table 3).

## 3 Outlook and Conclusion

Promising outcomes of patients with various kinds of solid tumors under ICB therapy have been seen in a substantial number of studies. Such great success spawns a series of anti-PD-1/PD-L1 agents that have been approved in clinics, among which pembrolizumab and atezolizumab have been approved for TNBC treatment globally. However, the hypoxic TME, is common in many solid tumors, including breast cancer, resulting in compromised efficacy. Therefore, combinatorial strategies of various kinds of systematic and local treatments that potentially arouse the abscopal effect with anti-PD-1/PD-L1 have attracted much attention in recent years. In addition to the combination strategies mentioned in the present review, there are still many other synergistic patterns that are at a very early phase of exploration. The antitumor adaptive immune response involves the cooperation of a series of immune cells, coexpressed molecules, cytokines, chemokines, etc. Given the complex characteristics of the immune response, it is insufficient to block PD-1/PD-L1 signaling alone. Preclinical research observed a synergistic effect by combining anti-CTLA-4 or other coinhibitory receptor blockades with PD-1 blockade (Herrera-Camacho et al., 2019; Jiang et al., 2021a). By combining hindering the coinhibitory signals, it has more potential to activate T cells at a higher level, which in turn initiates a stronger tumor-cell killing response (Hodi et al., 2016; Larkin et al., 2019). TGF-β has been confirmed to inhibit the immune response by inducing the differentiation of Tregs and antagonizing the function of immune cells, including T cells, APCs, and NK cells. Exciting results of anti-TGF-β plus anti-PD-1/PD-L1 have been reported in both preclinical and clinical (Chen et al., 2018; Tauriello et al., 2018). Since substantial cytokines are involved in the immune response, combining blockade of the immune checkpoint receptor and neutralization of costimulatory cytokines provides promising access to excite an antitumor immune response (Tsukamoto et al., 2018; Waldmann, 2018). The efficacy and safety of a few cytokine inhibitors plus ICB have been validated in clinics (Atkins et al., 2018; Wrangle et al., 2018; Algazi et al., 2020). Moreover, the gut microbiota attracts much attention in the field of host immune response. The function of providing the host with immune homeostasis and coordinating gut-associated immune cells plays a significant role in T cell response regulation (Pickard et al., 2017; Zhou et al., 2021a). Modification of the microbiota has been demonstrated to play a positive role in supplying intestinal macrophages by regulating the recruitment of circulating monocytes, which potentially participate in the inflammatory response (Amoroso et al., 2020) and tumorigenesis (Jia et al., 2018; Meng et al., 2018).

Combination therapy with PD-1/PD-L1 blockade has been highlighted in the medical field and extensively evaluated in breast cancer clinical trials. Trials investigating the efficacy and safety of combination therapy show promise for the benefit of combining PD-1/PD-L1 blockade with chemotherapy in both metastatic and early-stage disease settings (Table 2). Despite positive findings presented in preclinical studies, the clinical outcomes of several combination therapies in breast cancer patients have been disappointing. It is still unclear how to select appropriate combination therapy and identify biomarkers predicting the responses to combination therapy. The dosing schedule and timing and sequence of combination treatments should be optimized in the administration regimen, which directly influences the therapeutic outcome.

PD-L1 positivity and/or TILs are insufficient for patient selection for combinatorial therapy in breast cancer. Patient immune profiling and other predictive biomarkers can provide reasonable guidance for personalized combination therapy, which is helpful to optimize clinical benefits and minimize the cost of health care. A comprehensive framework integrating multimodal features, such as the genome, epigenome, transcriptome, proteome, and even metabolome, should be adopted to select patients benefiting from combination therapy.
