# Supplementary material for: Combinatorial Strategies With PD-1/PD-L1 Immune Checkpoint Blockade for Breast Cancer Therapy: Mechanisms and Clinical Outcomes
Source: Front Pharmacol. 2022 Jul 22;13:928369. doi: 10.3389/fphar.2022.928369 (PMC9355550; doi:10.3389/fphar.2022.928369)
Supplement: Supplementary file 1 [file DataSheet2.pdf]

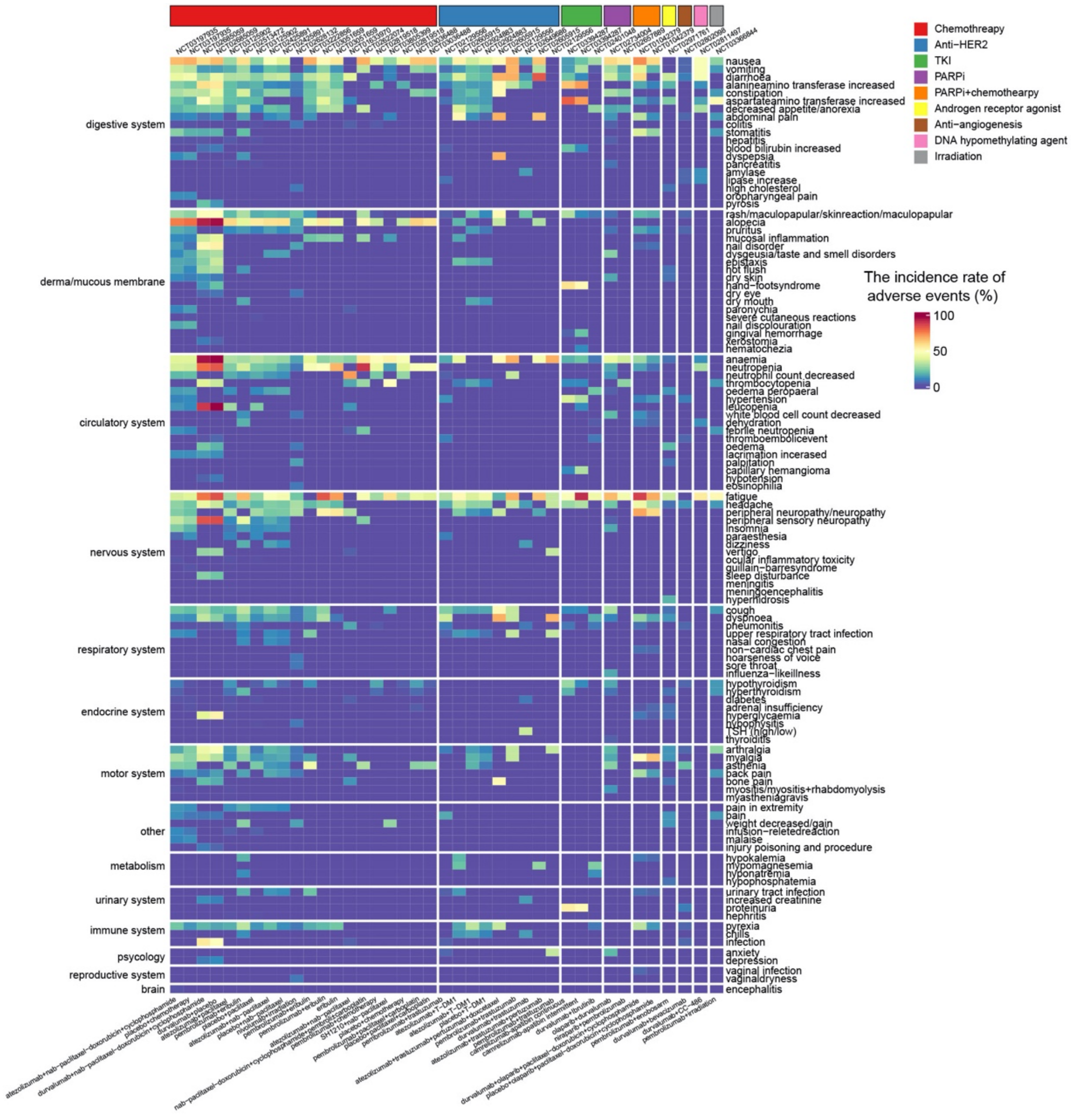

Supplementary figure 1. Heatmap showing the incidence of adverse events induced by combinatorial regimens of PD-1/PD-L1 blockades. The regimen of nab-paclitaxel-doxorubicin+cyclophosphamide was nab-paclitaxel followed by doxorubicin and cyclophosphamide. The color gradient shows the incidence of adverse events, where red and blue colors indicate the high and low rates.

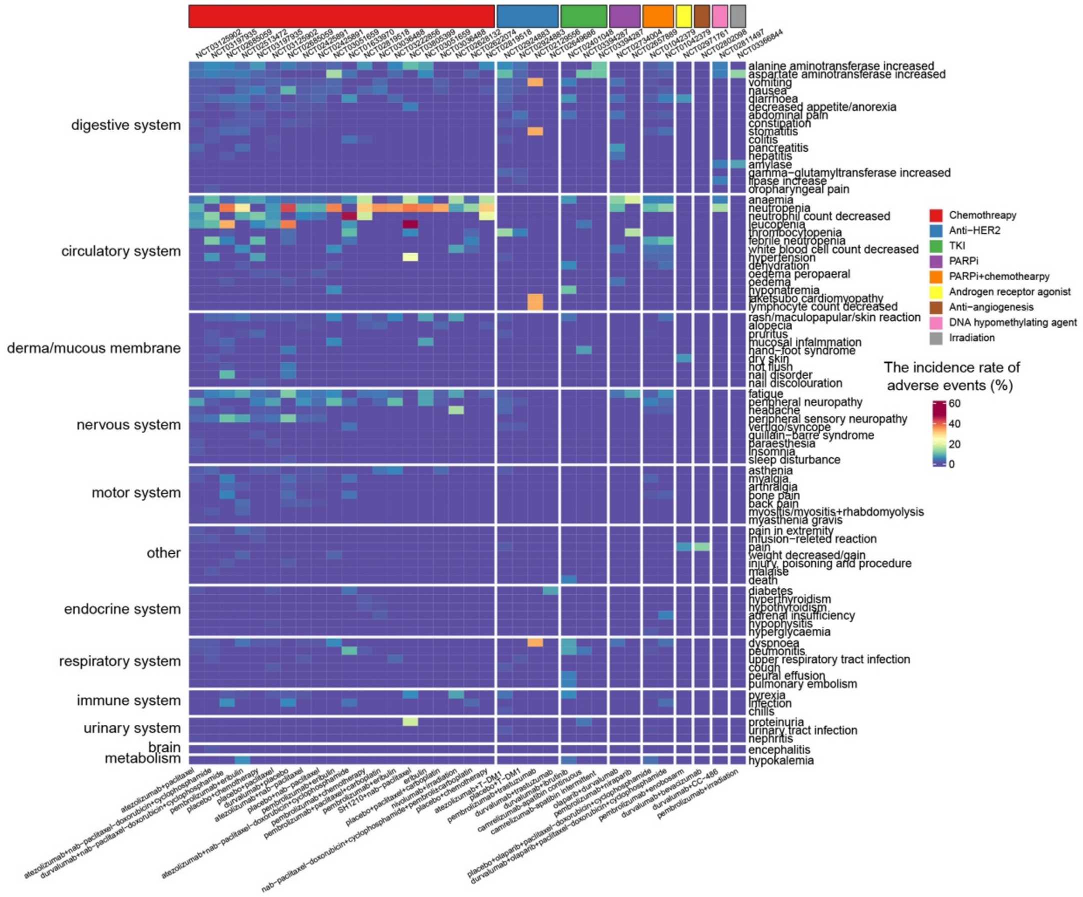

Supplementary figure 2. Heatmap showing the incidence rate of serious adverse events induced by combinatorial regimens of PD-1/PD-L1 blockades. The regimen of nab-paclitaxel-doxorubicin+cyclophosphamide was nab-paclitaxel followed by doxorubicin and cyclophosphamide. The color gradient shows the incidence of adverse events, where red and blue colors indicate the high and low rates.
