# Supplementary material for: Combinatorial Strategies With PD-1/PD-L1 Immune Checkpoint Blockade for Breast Cancer Therapy: Mechanisms and Clinical Outcomes
Source: Front Pharmacol. 2022 Jul 22;13:928369. doi: 10.3389/fphar.2022.928369 (PMC9355550; doi:10.3389/fphar.2022.928369)
Supplement: Supplementary file 2 [file DataSheet1.docx]

**Table S1 Clinical trials evaluating PD-1/PDL1 inhibitors in combination with other therapy strategies in breast cancer**

| **Combination strategy** | **NCT** | **Phase** | **Drug** | **Regimen** | **Additional therapy** | **ITT** |
| --- | --- | --- | --- | --- | --- | --- |
| Dual-drug | NCT02628132 | I/II | durvalumab 750mg, d1,15 | paclitaxel 80mg/m2, d1,8,15 | Chemotherapy | 21 (Ghebeh et al., 2021) |
| Dual-drug | NCT03805399 | Ib/II | SH1210 | nab-paclitaxel | Chemotherapy | 19 (Jiang et al., 2021) |
| Dual-drug | NCT02513472 | Ib/II | pembrolizumab 200mg d1, q3w | eribulin 1.4mg/m2, d1,8, q3w | Chemotherapy | 167 (Tolaney et al., 2021) |
| Dual-drug | NCT03051659 | II | pembrolizumab 200mg d1, q3w | eribulin 1.4mg/m2, d1,8, q3w | Chemotherapy | 90 (Tolaney et al., 2020) |
| Dual-drug | NCT03222856 | II | pembrolizumab 200mg d1, q3w | eribulin 1.23mg/m2, d1,8, q3w | Chemotherapy | 44 (Perez-Garcia et al., 2021) |
| Dual-drug | NCT02425891 | III | atezolizumab 840mg d1,15 | nab-Paclitaxel 100mg/m2, d1,8,15 | Chemotherapy | 902 (Schmid et al., 2018) |
| Dual-drug | NCT03197935 | III | atezolizumab 840mg d1,15 | nab-paclitaxel 125mg/m2, q1w | Chemotherapy | 333 (Mittendorf et al., 2020) |
| Dual-drug | NCT03125902 | III | atezolizumab 840mg d1,15 | paclitaxel 90mg/m2, d1,8,15 | Chemotherapy | 651 (Miles et al., 2021) |
| Dual-drug | NCT01633970 | Ib | atezolizumab 800mg d1,15 | nab-paclitaxel 125mg/m2, d1,8,15 | Chemotherapy | 33 (Adams et al., 2019) |
|  |  |  |  |  |  |  |
| Dual-drug | NCT03366844 | II | pembrolizumab 200mg, d2-7 | palliative Radiotherapy 4Gy × 5 | Irradiation | 8 (Barroso-Sousa et al., 2020) |
| Dual-drug | NCT02499367 | II | nivolumab q2w | 1) irradiation 8Gy × 3, 2) cyclophosphamide 50mg, qd, 3) cisplatin 40mg/m2, 4) doxorubcin 15mg/m2 | Irradiation, Chemotherapy | 70 (Voorwerk et al., 2019) |
| Dual-drug | NCT02605915 | Ib | atezolizumab 1200mg | T-DM1 3.6mg/Kg, trastuzumab(6mg/Kg,8mg/Kg), partuzumab (loading 840mg, maintenance 420 mg), docetaxel 75mg/m2 | Anti-HER2 | 73 (Hamilton et al., 2021) |
| Dual-drug | NCT02649686 | Ib | durvalumab 1125mg, d1 | trastuzumab 8mg/Kg loading, followed 6 mg/Kg, q3w | Anti-HER2 | 15 (Chia et al., 2019) |
| Dual-drug | NCT02129556 | Ib/II | pembrolizumab 2mg/Kg, 10mg/Kg, q3w | trastuzumab 6 mg/Kg | Anti-HER2 | 6 Ib, 52 II (Loi et al., 2019) |
| Dual-drug | NCT02924883 | II | atezolizumab 1200mg | T-DM1 3.6mg/Kg | Anti-HER2 | 202 |
| Dual-drug | NCT02802098 | I | durvalumab 10mg/Kg | bevacizumab 10mg/Kg q2w | Anti-angiogenesis | 26 (Quintela-Fandino et al., 2020) |
| Dual-drug | NCT03394287 | II | camrelizumab 200mg q2w | apatinib 250mg, continuous: d1-14, intermittent: d1-7 | TKI | 40 (Liu et al., 2020) |
| Dual-drug | NCT02401048 | Ib/II | durvalumab 10mg/Kg | ibrutinib 560 mg, daily | TKI | 45 (Hong et al., 2019) |
| Dual-drug | NCT02811497 | II | durvalumab 1500mg, d15 | CC-486 300 mg, d1-d14, 100 mg, qd, d1-d21 | DNA hypomethylating agent | 28 (Taylor et al., 2020) |
| Dual-drug | NCT02971761 | II | pembrolizumab 200mg d1, q3w | enobosarm 18mg, daily | Androgen receptor agonist | 18 (Yuan et al., 2021) |
| Dual-drug | NCT02734004 | I/II | durvalumab 1500mg, q4w | olaparib 300mg, twice daily | PARPi | 30 (Domchek et al., 2020) |
| Dual-drug | NCT02657889 | II | Pembrolizumab 200mg, d1, q3w | Niraparib 200mg, twice daily | PARPi | 55 (Vinayak et al., 2019) |
| Multi-drug | NCT02489448 | I/II | durvalumab 3mg/Kg, 10mg/Kg | nab-Paclitaxel 100 mg/m2-doxorubcin 60mg/m2+cyclophosphamide 600 mg/m2 | Chemotherapy | 45 (Ahmed et al., 2020) |
| Multi-drug | NCT02622074 | Ib | pembrolizumab 200mg | 1) nab-paclitaxel – doxorubicin + cyclophosphamide 2) nab-paclitaxel + carboplatin – doxorubicin + cyclophosphamide | Chemotherapy | 60 (Schmid et al., 2020b) |
| Multi-drug | NCT02685059 | II | durvalumab window: 750mg, 1500mg q4w | nab-paclitaxel 125mg/m2, weekly, 12w, followed by epirubicin (90 mg/m2) +cyclophosphamide (600mg/m2) | Chemotherapy | 174 (Loibl et al., 2019) |
| Multi-drug | NCT02819518 | III | pembrolizumab | nab-paclitaxel 100mg/m2, d1,8,15; Paclitaxel 90mg/m2, d1,8,15; or gemcitabine 1000 mg/m2 plus carboplatin AUC =2, d1.8 | Chemotherapy | 847 (Cortes et al., 2020) |
| Multi-drug | NCT03036488 | III | pembrolizumab 200mg, q3w | carboplatin AUC 5+paclitaxel 80mg/m2, q3w-doxorubcin 60mg/m2+cyclophosphamide 600 mg/m2 | Chemotherapy | 1174 (Schmid et al., 2020a) |
| Multi-drug | NCT01042379 | II/III | durvalumab | plaparib + paclitaxel (80mg/m2)-doxorubicin (60mg/m2) +cyclophosphamide (600mg/m2), trastuzumab 4mg/Kg loading, followed 2mg/Kg (for HER2+) | PARPi +Chemotherapy | 409 (Pusztai et al., 2021) |

Abbreviations: *ITT* intention to treat population.

**Table S2. Clinical trial results of PD-1/PD-L1 blockade in combination with other cancer treatment regimens**

| **Research** | **Subtype** | **Group** | **PP(n)** | **Results** |
| --- | --- | --- | --- | --- |
| NCT02628132 (Ghebeh et al., 2021) | TNBC | durvalumab + paclitaxel | 21 | ORR: 26% DCR: 47% CR: 5% mPFS: 4.0 m mOS: 20.7 m |
| NCT03805399 (Jiang et al., 2021) | TNBC | SH1210 + nab-paclitaxel | 19 | ORR: 62.5% |
| NCT02513472 (Tolaney et al., 2021) | TNBC | pembrolizumab + eribulin | 167 | ORR: 25.8 phase I, 21.8% phase II |
| NCT03051659 (Tolaney et al., 2020) | HR(+)HER2(-) | pembrolizumab + eribulin | 44 | mOS: 12.5 m mPFS: 4.1 m, ORR: 34.0%, PR+SD: 70.0% DOR: 1.5 m |
|  | HR(+)HER2(-) | eribulin | 46 | mOS: 13.4 m mPFS: 4.2 m, ORR: 27.0%, PR+SD: 70.0% DOR: 2.1 m |
| NCT03222856 (Perez-Garcia et al., 2021) | HR(+)HER2(-) | pembrolizumab + eribulin | 44 | mPFS: 6.0 m 1y-OS: 59.1% ORR: 40.9% DCR: 25% |
| NCT02425891 (Schmid et al., 2018) | TNBC | atezolizumab + nab-paclitaxel | 451 | mOS: 21.3 m,  mPFS: 7.2 m, 1y-PFS: 23.7%(19.6-27.9),  2y-PFS: 42.1%(34.3-49.9),  pCR:7.1%,  ORR: 56.0%, DOR: 7.4 m |
|  | TNBC | placebo + nab-paclitaxel | 451 | mOS: 17.6 m, mPFS: 5.5 m, 1y-PFS: 17.7% (14.0-21.4),  2y-PFS: 39.7%(33.2-46.3),  pCR:1.6%,  ORR: 45.9%, DOR: 5.6 m |
| NCT03197935 (Mittendorf et al., 2020) | TNBC | atezolizumab + nab-paclitaxel-doxorubicin + cyclophosphamide) | 165 | pCR:58.0% |
|  | TNBC | placebo + chemotherapy | 168 | pCR:41.0% |
| NCT03125902 (Miles et al., 2021) | TNBC | atezolizumab + paclitaxel | 431 | mOS: 19.2 m,  mPFS: 5.9 m, 1y-OS: 69% (64-73),  2y-OS: 42% (36-48),  pCR: 5.0%,  ORR: 43.0%, DOR: 7.7m |
|  | TNBC | placebo + paclitaxel | 220 | mOS: 22.8 m,  mPFS: 5.6 m, 1y-OS: 73% (67-79),  2y-OS: 45% (36-54),  pCR: 5.0%,  ORR: 36.0%, DOR: 5.8 m |
| NCT01633970 (Adams et al., 2019) | TNBC | atezolizumab + nab-paclitaxel | 33 | ORR: 39.4%  CR: 3.0%  DCR: 51.5%  mDOR: 9.1m  mPFS: 5.5m  mOS: 14.7m |
| NCT03366844 (Barroso-Sousa et al., 2020) | HR(+) HER2(-) | pembrolizumab + irradiation | 8 | mOS: 2.9 m mPFS: 1.4 m |
| NCT02499367 (Voorwerk et al., 2019) | TNBC | nivolumab | 12 | ORR: 17% |
|  | TNBC | nivolumab + irradiation | 12 | ORR: 8% |
|  | TNBC | nivolumab + cisplatin | 13 | ORR: 23% |
|  | TNBC | nivolumab + doxorubicin | 17 | ORR: 35% |
|  | TNBC | nivolumab + cyclophosphamide | 12 | ORR: 8% |
| NCT02605915 (Hamilton et al., 2021) | HER(+) | atezolizumab + trastuzumab + pertuzumab | mBC: 6, eBC: 20 | ORR: 14% (mBC), 65% (eBC) |
|  | HER(+) | atezolizumab + T-DM1 | mBC: 6, eBC: 20 | ORR: 35% (mBC), 70% (eBC) |
|  | HER(+) | atezolizumab + trastuzumab + pertuzumab + doxcetaxel | mBC: 6 | ORR: 100% (mBC) |
| NCT02649686 (Chia et al., 2019) | HER2(+) | durvalumab + trastuzumab | 15 | SD: 29% DOR: 2.7 m mPFS: 1.35 m 6-month PFS: 0 6-month OS: 51.6% 1-year OS: 17.2% |
| NCT02129556 (Loi et al., 2019) | HER2(+) | pembrolizumab + trastuzumab | 58 | PD-L1-positive:  ORR: 15% DCR: 11% (24w) CR: 4% |
| NCT02924883 (Emens et al., 2020) | HER2(+) | atezolizumab + T-DM1 | 133 | mPFS: 8.2 m, pCR: 6.0%,  ORR: 45.0%, DOR: 7.1 m |
|  | HER2(+) | placebo + T-DM1 | 69 | mPFS: 6.8 m, pCR: 7.2%,  ORR: 43.0%, DOR: 9.9 m |
| NCT02802098 (Quintela-Fandino et al., 2020) | HER(-) | durvalumablumab + bevacizumab | 25 | mOS: 11.0 m mPFS: 3.5 m DCR: 60% (at 8w), 44% (at 16w) |
| NCT03394287 (Liu et al., 2020) | TNBC | camrelizumab + apatinib continuous | 30 | mOS: 8.1 m mPFS: 3.7 m, 1y-OS: 42.2% (24.2-59.2),  ORR: 43.3%, DCR: 63.3% DOR: 6.6 m |
|  | TNBC | camrelizumab + apatinib intermittent | 10 | mOS: 9.5 m mPFS: 1.9 m, 1y-OS: 40.0% (12.3-67),  ORR: 0, DCR: 40.0% DOR: 1.9 m |
| NCT02401048 (Hong et al., 2019) | TNBC/HER2(+) | durvalumab + ibrutinib | 45 | mOS: 4.2 m mPFS: 1.7 m ORR: 3% |
| NCT02811497 (Taylor et al., 2020) | ER(+)HER2(-) | durvalumab + CC-486 (300mg, q1-14) | 28 | mPFS: 1.9 m mOS: 5.0 m DCR: 7.1% |
| NCT02971761 (Yuan et al., 2021) | TNBC | pembrolizumab + enobosarm | 16 | mOS: 25.5 m mPFS: 2.6 m ORR: 13% CR: 6% DCR: 25% (16w) |
| NCT02734004 (Domchek et al., 2020) | HER2(-) | olaparib + durvalumab | 24 | mOS: 21.5 m mPFS: 8.2 m  DCR: 80.0% (at 12w), 50.0% (at 28w) ORR: 63.3% (at 12w) CR: 3% |
| NCT02657889 (Vinayak et al., 2019) | TNBC | Niraparib+pembrolizumab | 47 | ORR:18.2%  CR:9.1%  DCR:41.8%  mPFS: 2.3m  6m-PFS: 28%  12m-PFS: 14% |
| NCT02489448 (Ahmed et al., 2020) | TNBC | nab-paclitaxel + durvalumab- epirubicin + cyclophosphamide + durvalumab | 45 | pCR: 40% |
| NCT02622074 (Schmid et al., 2020b) | TNBC | nab-paclitaxel-doxorubicin + cyclophosphamide + pembrolizumab ± carboplatin | 60 | pCR: ypT0/Tis ypN0 60%, ypT0 ypN0 57% |
| NCT02685059 (Loibl et al., 2019) | TNBC | durvalumab + NACT (nab-paclitaxel-epirubicin + cyclophosphamide) | 88 | pCR: 53.4% |
|  | TNBC | durvalumab + placebo | 86 | pCR: 44.2% |
| NCT02819518 (Cortes et al., 2020) | TNBC | pembrolizumab + chemotherapy | 566 | CPS≥10: mPFS 9.7 m CPS ≥1: mPFS 7.6 m ITT mPFS 7.5 m |
|  | TNBC | placebo + chemotherapy | 281 | CPS≥10: mPFS 5.6 m CPS ≥1: mPFS 5.6 m ITT mPFS 5.6 m |
| NCT03036488 (Schmid et al., 2020a) | TNBC | pembrolizumab + paclitaxel + carboplatin | 784 | pCR: 64.8% |
|  | TNBC | placebo + paclitaxel + carboplatin | 390 | pCR: 51.2% |
| NCT01042379 (Pusztai et al., 2021) | HER(-) | placebo + olaparib + paclitaxel-doxorubicin + cyclophosphamide | 229 | pCR: 20% HER2-, 14% HR+HER-, 27% TNBC |
|  | HER(-) | durvalumab + olaparib + paclitaxel-doxorubicin + cyclophosphamide | 73 | pCR: 37% HER2-, 28% HR+HER-, 47% TNBC |

Abbreviations: *PP* per protocol population, *pCR* pathological complete response, *ORR* objective response rate, *DCR* disease control rate, *CR* complete response, *mPFS* median progression-free survival, *mOS* median overall survival, *PR* partially response, *SD* stable disease, *DOR* during of response, *NACT* neoadjuvant chemotherapy, *TNBC* triple-negative breast cancer, *HER2* human epidermal growth factor receptor-2, *HR* hormonal receptor.

**Reference**

Adams, S., Diamond, J. R., Hamilton, E., Pohlmann, P. R., Tolaney, S. M., Chang, C. W., et al. (2019). Atezolizumab Plus nab-Paclitaxel in the Treatment of Metastatic Triple-Negative Breast Cancer With 2-Year Survival Follow-up: A Phase 1b Clinical Trial. *JAMA Oncol,* 5**,** 334-342. <https://doi.org/10.1001/jamaoncol.2018.5152>

Ahmed, F. S., Gaule, P., Mcguire, J., Patel, K., Blenman, K., Pusztai, L., et al. (2020). PD-L1 Protein Expression on Both Tumor Cells and Macrophages are Associated with Response to Neoadjuvant Durvalumab with Chemotherapy in Triple-negative Breast Cancer. *Clin Cancer Res,* 26**,** 5456-5461. <https://doi.org/10.1158/1078-0432.CCR-20-1303>

Barroso-Sousa, R., Krop, I. E., Trippa, L., Tan-Wasielewski, Z., Li, T., Osmani, W., et al. (2020). A Phase II Study of Pembrolizumab in Combination With Palliative Radiotherapy for Hormone Receptor-positive Metastatic Breast Cancer. *Clin Breast Cancer,* 20**,** 238-245. <https://doi.org/10.1016/j.clbc.2020.01.012>

Chia, S., Bedard, P. L., Hilton, J., Amir, E., Gelmon, K., Goodwin, R., et al. (2019). A Phase Ib Trial of Durvalumab in Combination with Trastuzumab in HER2-Positive Metastatic Breast Cancer (CCTG IND.229). *Oncologist,* 24**,** 1439-1445. <https://doi.org/10.1634/theoncologist.2019-0321>

Cortes, J., Cescon, D. W., Rugo, H. S., Nowecki, Z., Im, S. A., Yusof, M. M., et al. (2020). Pembrolizumab plus chemotherapy versus placebo plus chemotherapy for previously untreated locally recurrent inoperable or metastatic triple-negative breast cancer (KEYNOTE-355): a randomised, placebo-controlled, double-blind, phase 3 clinical trial. *Lancet,* 396**,** 1817-1828. <https://doi.org/10.1016/S0140-6736(20)32531-9>

Domchek, S. M., Postel-Vinay, S., Im, S. A., Park, Y. H., Delord, J. P., Italiano, A., et al. (2020). Olaparib and durvalumab in patients with germline BRCA-mutated metastatic breast cancer (MEDIOLA): an open-label, multicentre, phase 1/2, basket study. *Lancet Oncol,* 21**,** 1155-1164. <https://doi.org/10.1016/S1470-2045(20)30324-7>

Emens, L. A., Esteva, F. J., Beresford, M., Saura, C., De Laurentiis, M., Kim, S. B., et al. (2020). Trastuzumab emtansine plus atezolizumab versus trastuzumab emtansine plus placebo in previously treated, HER2-positive advanced breast cancer (KATE2): a phase 2, multicentre, randomised, double-blind trial. *Lancet Oncol,* 21**,** 1283-1295. <https://doi.org/10.1016/S1470-2045(20)30465-4>

Ghebeh, H., Al-Sayed, A., Eiada, R., Cabangon, L., Ajarim, D., Suleman, K., et al. (2021). Weekly Paclitaxel given concurrently with Durvalumab has a favorable safety profile in triple-negative metastatic breast cancer. *Sci Rep,* 11**,** 19154. <https://doi.org/10.1038/s41598-021-98113-6>

Hamilton, E. P., Kaklamani, V., Falkson, C., Vidal, G. A., Ward, P. J., Patre, M., et al. (2021). Impact of Anti-HER2 Treatments Combined With Atezolizumab on the Tumor Immune Microenvironment in Early or Metastatic Breast Cancer: Results From a Phase Ib Study. *Clin Breast Cancer,* 21**,** 539-551. <https://doi.org/10.1016/j.clbc.2021.04.011>

Hong, D., Rasco, D., Veeder, M., Luke, J. J., Chandler, J., Balmanoukian, A., et al. (2019). A Phase 1b/2 Study of the Bruton Tyrosine Kinase Inhibitor Ibrutinib and the PD-L1 Inhibitor Durvalumab in Patients with Pretreated Solid Tumors. *Oncology,* 97**,** 102-111. <https://doi.org/10.1159/000500571>

Jiang, Y. Z., Liu, Y., Xiao, Y., Hu, X., Jiang, L., Zuo, W. J., et al. (2021). Molecular subtyping and genomic profiling expand precision medicine in refractory metastatic triple-negative breast cancer: the FUTURE trial. *Cell Res,* 31**,** 178-186. <https://doi.org/10.1038/s41422-020-0375-9>

Liu, J., Liu, Q., Li, Y., Li, Q., Su, F., Yao, H., et al. (2020). Efficacy and safety of camrelizumab combined with apatinib in advanced triple-negative breast cancer: an open-label phase II trial. *J Immunother Cancer,* 8. <https://doi.org/10.1136/jitc-2020-000696>

Loi, S., Giobbie-Hurder, A., Gombos, A., Bachelot, T., Hui, R., Curigliano, G., et al. (2019). Pembrolizumab plus trastuzumab in trastuzumab-resistant, advanced, HER2-positive breast cancer (PANACEA): a single-arm, multicentre, phase 1b-2 trial. *Lancet Oncol,* 20**,** 371-382. <https://doi.org/10.1016/S1470-2045(18)30812-X>

Loibl, S., Untch, M., Burchardi, N., Huober, J., Sinn, B. V., Blohmer, J. U., et al. (2019). A randomised phase II study investigating durvalumab in addition to an anthracycline taxane-based neoadjuvant therapy in early triple-negative breast cancer: clinical results and biomarker analysis of GeparNuevo study. *Ann Oncol,* 30**,** 1279-1288. <https://doi.org/10.1093/annonc/mdz158>

Miles, D., Gligorov, J., Andre, F., Cameron, D., Schneeweiss, A., Barrios, C., et al. (2021). Primary results from IMpassion131, a double-blind, placebo-controlled, randomised phase III trial of first-line paclitaxel with or without atezolizumab for unresectable locally advanced/metastatic triple-negative breast cancer. *Ann Oncol,* 32**,** 994-1004. <https://doi.org/10.1016/j.annonc.2021.05.801>

Mittendorf, E. A., Zhang, H., Barrios, C. H., Saji, S., Jung, K. H., Hegg, R., et al. (2020). Neoadjuvant atezolizumab in combination with sequential nab-paclitaxel and anthracycline-based chemotherapy versus placebo and chemotherapy in patients with early-stage triple-negative breast cancer (IMpassion031): a randomised, double-blind, phase 3 trial. *Lancet,* 396**,** 1090-1100. <https://doi.org/10.1016/S0140-6736(20)31953-X>

Perez-Garcia, J. M., Llombart-Cussac, A., M, G. C., Curigliano, G., Lopez-Miranda, E., Alonso, J. L., et al. (2021). Pembrolizumab plus eribulin in hormone-receptor-positive, HER2-negative, locally recurrent or metastatic breast cancer (KELLY): An open-label, multicentre, single-arm, phase trial. *Eur J Cancer,* 148**,** 382-394. <https://doi.org/10.1016/j.ejca.2021.02.028>

Pusztai, L., Yau, C., Wolf, D. M., Han, H. S., Du, L., Wallace, A. M., et al. (2021). Durvalumab with olaparib and paclitaxel for high-risk HER2-negative stage II/III breast cancer: Results from the adaptively randomized I-SPY2 trial. *Cancer Cell,* 39**,** 989-998 e5. <https://doi.org/10.1016/j.ccell.2021.05.009>

Quintela-Fandino, M., Holgado, E., Manso, L., Morales, S., Bermejo, B., Colomer, R., et al. (2020). Immuno-priming durvalumab with bevacizumab in HER2-negative advanced breast cancer: a pilot clinical trial. *Breast Cancer Res,* 22**,** 124. <https://doi.org/10.1186/s13058-020-01362-y>

Schmid, P., Adams, S., Rugo, H. S., Schneeweiss, A., Barrios, C. H., Iwata, H., et al. (2018). Atezolizumab and Nab-Paclitaxel in Advanced Triple-Negative Breast Cancer. *N Engl J Med,* 379**,** 2108-2121. <https://doi.org/10.1056/NEJMoa1809615>

Schmid, P., Cortes, J., Pusztai, L., Mcarthur, H., Kummel, S., Bergh, J., et al. (2020a). Pembrolizumab for Early Triple-Negative Breast Cancer. *N Engl J Med,* 382**,** 810-821. <https://doi.org/10.1056/NEJMoa1910549>

Schmid, P., Salgado, R., Park, Y. H., Munoz-Couselo, E., Kim, S. B., Sohn, J., et al. (2020b). Pembrolizumab plus chemotherapy as neoadjuvant treatment of high-risk, early-stage triple-negative breast cancer: results from the phase 1b open-label, multicohort KEYNOTE-173 study. *Ann Oncol,* 31**,** 569-581. <https://doi.org/10.1016/j.annonc.2020.01.072>

Taylor, K., Loo Yau, H., Chakravarthy, A., Wang, B., Shen, S. Y., Ettayebi, I., et al. (2020). An open-label, phase II multicohort study of an oral hypomethylating agent CC-486 and durvalumab in advanced solid tumors. *J Immunother Cancer,* 8. <https://doi.org/10.1136/jitc-2020-000883>

Tolaney, S. M., Barroso-Sousa, R., Keenan, T., Li, T., Trippa, L., Vaz-Luis, I., et al. (2020). Effect of Eribulin With or Without Pembrolizumab on Progression-Free Survival for Patients With Hormone Receptor-Positive, ERBB2-Negative Metastatic Breast Cancer: A Randomized Clinical Trial. *JAMA Oncol,* 6**,** 1598-1605. <https://doi.org/10.1001/jamaoncol.2020.3524>

Tolaney, S. M., Kalinsky, K., Kaklamani, V. G., D'adamo, D. R., Aktan, G., Tsai, M. L., et al. (2021). Eribulin Plus Pembrolizumab in Patients with Metastatic Triple-Negative Breast Cancer (ENHANCE 1): A Phase Ib/II Study. *Clin Cancer Res,* 27**,** 3061-3068. <https://doi.org/10.1158/1078-0432.CCR-20-4726>

Vinayak, S., Tolaney, S. M., Schwartzberg, L., Mita, M., Mccann, G., Tan, A. R., et al. (2019). Open-label Clinical Trial of Niraparib Combined With Pembrolizumab for Treatment of Advanced or Metastatic Triple-Negative Breast Cancer. *JAMA Oncol,* 5**,** 1132-1140. <https://doi.org/10.1001/jamaoncol.2019.1029>

Voorwerk, L., Slagter, M., Horlings, H. M., Sikorska, K., Van De Vijver, K. K., De Maaker, M., et al. (2019). Immune induction strategies in metastatic triple-negative breast cancer to enhance the sensitivity to PD-1 blockade: the TONIC trial. *Nat Med,* 25**,** 920-928. <https://doi.org/10.1038/s41591-019-0432-4>

Yuan, Y., Lee, J. S., Yost, S. E., Frankel, P. H., Ruel, C., Egelston, C. A., et al. (2021). A Phase II Clinical Trial of Pembrolizumab and Enobosarm in Patients with Androgen Receptor-Positive Metastatic Triple-Negative Breast Cancer. *Oncologist,* 26**,** 99-e217. <https://doi.org/10.1002/onco.13583>
